# The alternative serotonin transporter promoter P2 impacts gene function in females with irritable bowel syndrome

**DOI:** 10.1111/jcmm.16736

**Published:** 2021-06-24

**Authors:** Sandra Mohr, Nikola Fritz, Christian Hammer, Cristina Martínez, Sabrina Berens, Stefanie Schmitteckert, Verena Wahl, Malin Schmidt, Lesley A. Houghton, Miriam Goebel‐Stengel, Maria Kabisch, Dorothea Götze, Irina Milovač, Mauro D’Amato, Tenghao Zheng, Ralph Röth, Hubert Mönnikes, Felicitas Engel, Annika Gauss, Jonas Tesarz, Martin Raithel, Viola Andresen, Thomas Frieling, Jutta Keller, Christian Pehl, Christoph Stein‐Thöringer, Gerard Clarke, Paul J. Kennedy, John F. Cryan, Timothy G. Dinan, Eamonn M. M. Quigley, Robin Spiller, Caroll Beltrán, Ana María Madrid, Verónica Torres, Edith Pérez de Arce, Wolfgang Herzog, Emeran A. Mayer, Gregory Sayuk, Maria Gazouli, George Karamanolis, Lejla Kapur‐Pojskič, Mariona Bustamante, Raquel Rabionet, Xavier Estivil, André Franke, Wolfgang Lieb, Guy Boeckxstaens, Mira M. Wouters, Magnus Simrén, Gudrun A. Rappold, Maria Vicario, Javier Santos, Rainer Schaefert, Justo Lorenzo‐Bermejo, Beate Niesler

**Affiliations:** ^1^ Department of Human Molecular Genetics Institute of Human Genetics Heidelberg University Hospital Heidelberg Germany; ^2^ Department of Cancer Immunology Genentech South San Francisco CA USA; ^3^ Department of Human Genetics Genentech South San Francisco CA USA; ^4^ Institut de Recerca Biomèdica de Lleida (IRBLleida) Lleida Spain; ^5^ Lleida Institute for Biomedical Research Dr. Pifarré Foundation (IRBLleida) Lleida Spain; ^6^ Department of General Internal Medicine and Psychosomatics Heidelberg University Hospital Heidelberg Germany; ^7^ Department of Translational Brain Research Central Institute of Mental Health; ^8^ St. James’s University Hospital University of Leeds Leeds UK; ^9^ Mayo Clinic Jacksonville FL USA; ^10^ Department of Psychosomatic Medicine University Hospital Tübingen Tübingen Germany; ^11^ Department of Internal Medicine and Gastroenterology HELIOS Clinic Rottweil Rottweil Germany; ^12^ Institute of Medical Biometry and Informatics Heidelberg University Heidelberg Germany; ^13^ Faculty of Medicine University Banja Luka Banja Luka Bosnia and Herzegovina; ^14^ School of Biological Sciences Monash University Clayton VIC Australia; ^15^ Unit of Clinical Epidemiology Department of Medicine Solna Karolinska Institutet Stockholm Sweden; ^16^ Department of Gastrointestinal and Liver Diseases Biodonostia HRI San Sebastián Spain; ^17^ IKERBASQUE Basque Foundation for Science Bilbao Spain; ^18^ nCounter Core Facility Department of Human Molecular Genetics Heidelberg University Hospital Heidelberg Germany; ^19^ Martin‐Luther‐Hospital Berlin Germany; ^20^ Department of Gastroenterology, Infectious Diseases and Intoxications Heidelberg University Heidelberg Germany; ^21^ University of Erlangen Erlangen Germany; ^22^ Israelitisches Krankenhaus Hamburg Germany; ^23^ Helios Klinik Krefeld Krefeld Germany; ^24^ Krankenhaus Vilsbiburg Vilsbiburg Germany; ^25^ German Cancer Research Center Heidelberg Germany; ^26^ Department of Psychiatry and Neurobehavioral Science University College Cork Cork Ireland; ^27^ APC Microbiome Ireland University College Cork Cork Ireland; ^28^ Department of Anatomy and Neuroscience University College Cork Cork Ireland; ^29^ Lynda K. and David M. Underwood Center for Digestive Disorders Houston Methodist Hospital Weill Cornell Medical College Houston TX USA; ^30^ Nottingham Digestive Diseases Centre University of Nottingham Nottingham UK; ^31^ Gastroenterology Unit Hospital Clínico Universidad de Chile Medicine Department Universidad de Chile Santiago de Chile Chile; ^32^ Oppenheimer Center for Neurobiology of Stress University of California Los Angeles CA USA; ^33^ Washington University School of Medicine St. Louis MO USA; ^34^ Laboratory of Biology Medical School National and Kapodistrian University of Athens Athens Greece; ^35^ Academic Department of Gastroenterology Medical School National and Kapodistrian University of Athens "Laikon" General Hospital Athens Greece; ^36^ Institute for Genetic Engineering and Biotechnology University of Sarajevo Sarajevo Bosnia and Herzegovina; ^37^ CRG Centre for Genomic Regulation Barcelona Spain; ^38^ ISGlobal Barcelona Spain; ^39^ Department of Genetics, Microbiology and Statistics Faculty of Biology IBUB CIBERER IRSJD Universitat de Barcelona Barcelona Spain; ^40^ Institute of Clinical Molecular Biology Kiel Germany; ^41^ Institute of Epidemiology Kiel Germany; ^42^ TARGID University Hospital Leuven Leuven Belgium; ^43^ Institute of Medicine University of Gothenburg Gothenburg Sweden; ^44^ Interdisciplinary Center for Neurosciences (IZN) Heidelberg University Heidelberg Germany; ^45^ Institut de Recerca Vall d’Hebron Hospital Vall d'Hebron Barcelona Spain; ^46^ Department of Psychosomatic Medicine Division of Internal Medicine University Hospital Basel Basel Switzerland; ^47^ Faculty of Medicine University of Basel Basel Switzerland

**Keywords:** 5‐HT, IBS, IBS‐C, serotonin transporter

## Abstract

Irritable bowel syndrome (IBS) is a gut‐brain disorder in which symptoms are shaped by serotonin acting centrally and peripherally. The serotonin transporter gene *SLC6A4* has been implicated in IBS pathophysiology, but the underlying genetic mechanisms remain unclear. We sequenced the alternative P2 promoter driving intestinal *SLC6A4* expression and identified single nucleotide polymorphisms (SNPs) that were associated with IBS in a discovery sample. Identified SNPs built different haplotypes, and the tagging SNP rs2020938 seems to associate with constipation‐predominant IBS (IBS‐C) in females. rs2020938 validation was performed in 1978 additional IBS patients and 6,038 controls from eight countries. Meta‐analysis on data from 2,175 IBS patients and 6,128 controls confirmed the association with female IBS‐C. Expression analyses revealed that the P2 promoter drives *SLC6A4* expression primarily in the small intestine. Gene reporter assays showed a functional impact of SNPs in the P2 region. In silico analysis of the polymorphic promoter indicated differential expression regulation. Further follow‐up revealed that the major allele of the tagging SNP rs2020938 correlates with differential *SLC6A4* expression in the jejunum and with stool consistency, indicating functional relevance. Our data consolidate rs2020938 as a functional SNP associated with IBS‐C risk in females, underlining the relevance of *SLC6A4* in IBS pathogenesis.

## 1 INTRODUCTION

Accumulating evidence has shown that irritable bowel syndrome (IBS) is not just a functional gastrointestinal (GI) disorder, but rather represents a prototypical gut‐brain disorder.^1^, ^2^ IBS patients present with abdominal pain and variable alterations in bowel habits, which define the subtype of IBS. These subtypes are diarrhoea‐predominant IBS (IBS‐D), constipation‐predominant IBS (IBS‐C), mixed IBS (IBS‐M) or unspecified IBS (IBS‐U).^2^, ^3^ IBS is one of the common GI disorders, and depending on the applied diagnostic Rome criteria, it has a prevalence of 10.1% (Rome III) and 4.1% (Rome IV) worldwide,^4^ and 70%‐75% of affected individuals are female.^2^, ^5^

Bidirectional communication between the gut and the brain via the gut‐brain axis is influenced by the immune system, hormones and neurotransmitters.^2^, ^3^ Serotonin, or 5‐hydroxytryptamine (5‐HT), acts as a neurotransmitter, paracrine factor, endocrine hormone and growth factor to connect the gut and the brain.^6^ Enteric 5‐HT regulates a range of gut functions, including GI motility, secretion and visceral sensation.^6^ Of note, alterations in both central and peripheral 5‐HT contribute to visceral hypersensitivity in IBS.^7^ Furthermore, 5‐HT influences behaviour and modulates the immune and nervous systems. It affects the vagus nerve and is involved in the GI symptoms and comorbid disorders associated with IBS.^6^, ^8^, ^9^ In line with its important gut‐related functions, serotonin is predominantly produced in the gut and is influenced by gut microbiota.^10^

IBS is caused by extrinsic factors such as stress, infection and diet, as well as intrinsic factors including the individual genetic background and microbiota.^7^, ^11^ One of the most extensively analysed genes in IBS is *SLC6A4* (NM_001045), which encodes the serotonin re‐uptake transporter (SERT).^11^ This transporter is responsible for the re‐uptake of serotonin from the synaptic cleft into the presynaptic neuron and from the interstitial space into the gut epithelium and enterocytes.

**FIGURE 1:**
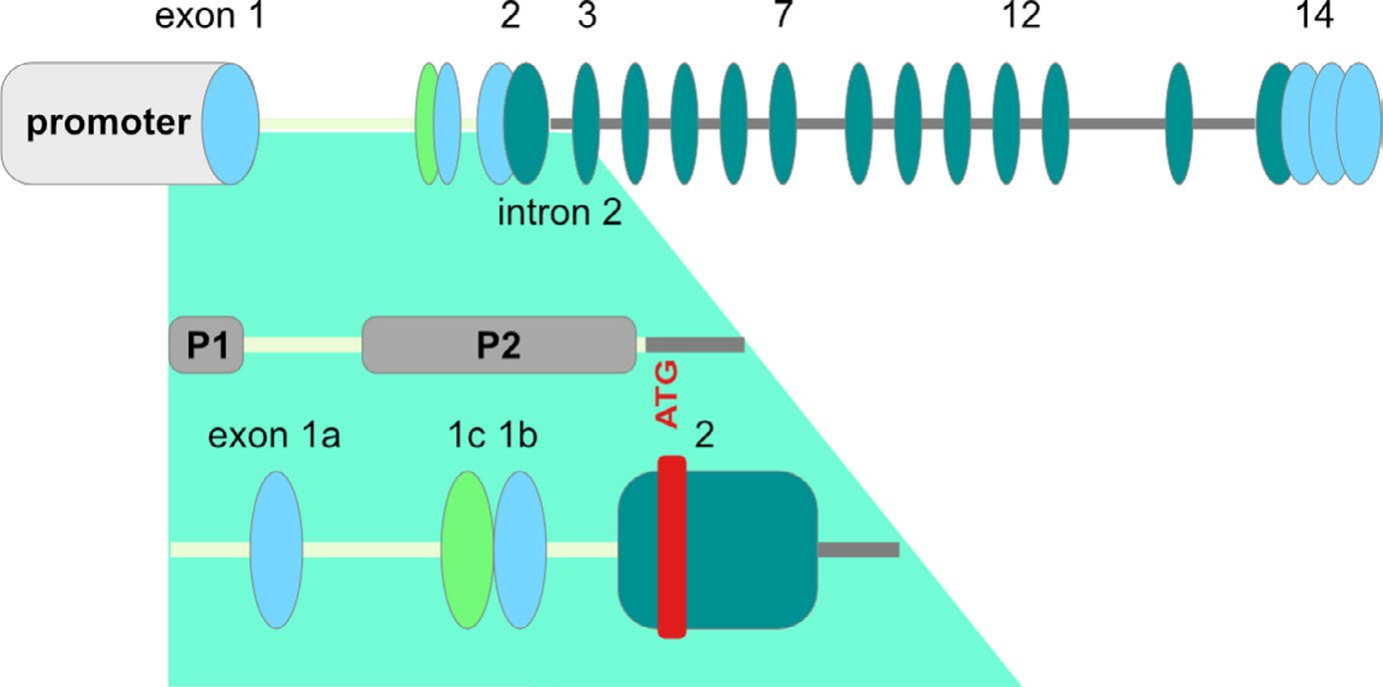
Schematic showing the upstream region of *SLC6A4* including the promoter regions P1 and P2. Oval shapes represent exons, and connecting lines indicate introns. Upstream regions of the start ATG (5’untranslated region [5’UTR]) are indicated by a light yellow line. Alternative upstream non‐coding exons are indicated in light blue/green and downstream coding exons are labelled in dark green. Exons 1a, 1b, 1c and 2, promoter 1 (P1) and the alternative promoter 2 (P2) are indicated (adapted from^14^). Not drawn to scale

The expression of different 5’ *SLC6A4* isoforms is driven by two distinct promoters—P1 and P2 ^12^, ^13^, ^14^ (Figure 1). P1 controls the expression of isoforms 1a and 1b, which have non‐coding exons representing alternatively used 5’ untranslated regions (5’UTRs) upstream of exon 2. A novel *SLC6A4* isoform is generated when exon 1c and 1b are spliced to exon 2, and expression of this isoform is driven by the P2 promoter downstream of P1 (Figure 1). This novel isoform is predominantly expressed in the GI tract.^14^

A 44‐bp insertion/deletion polymorphism, 5‐HTTLPR (short, long: s/l), in the P1 promoter of *SLC6A4* had been associated with IBS, although some studies have failed to confirm this association (for a summary, see^11^ ). The s allele of promoter P1 is a less potent driver of mRNA expression, so may diminish serotonin re‐uptake, leading to higher 5‐HT levels and increased bioavailability in the gut.^12^, ^15^ 5‐HT levels were higher in rectal biopsies of IBS‐D patients compared with biopsies from healthy individuals or individuals with IBS‐A or IBS‐C.^16^ These 5‐HT levels were significantly higher in carriers of the s/s genotype than in carriers of the s/l or l/l genotype.^16^ Increased 5‐HT levels and the s/s genotype correlated significantly with IBS‐D and abdominal pain,^16^ whereas the l/l genotype was associated with IBS‐C. The s/l genotype correlated with SERT protein expression (SERT expression was lower in colon biopsies of s allele carriers).^15^, ^16^

The P1 s/l polymorphism is also associated with behavioural traits and psychiatric conditions like anxiety and depression that are often comorbid with IBS, supporting the biopsychosocial model of IBS.^13^, ^17^, ^18^ Furthermore, stress is involved in shaping the phenotype.^19^, ^20^ These findings suggest that the P1 s/l polymorphism might alter central neurobiological functions in the brain to induce behavioural phenotypes.

Disturbed GI function is well documented in IBS, but the underlying neuromolecular mechanisms are not well understood. Serotonin metabolism is altered in IBS, and normalizing serotonin levels can ameliorate IBS symptoms.^2^, ^21^ Studies have revealed variable results regarding levels of 5‐HT and its metabolites in IBS,^15^, ^22^, ^23^, ^24^, ^25^ but SERT expression was consistently found reduced in GI tissues of IBS patients, which supports findings of increased 5‐HT in the tissue or blood.^22^, ^23^ How genetic factors affect 5‐HT in IBS is still poorly understood. Unravelling these genetic mechanisms may help to develop effective, individualized treatment strategies for IBS.

In this study, we aimed to elucidate the impact of the alternative P2 promoter of *SLC6A4* in IBS pathogenesis. We sequenced the P2 region of *SLC6A4* in a discovery sample from the United Kingdom and identified SNPs that were associated with IBS‐C in females. To validate the initial association of the SNPs with IBS‐C, we genotyped a tagging SNP in eleven cohorts from eight additional countries. Meta‐analysis confirmed the initial finding. Follow‐up analyses of associated variants were performed by luciferase reporter assays to assess their functional impact. Expression analysis in different GI regions uncovered the relevance of the P1‐ and P2‐driven isoforms in the gut. We also analysed the functional impact on promoter regulation *in* *silico*. The genotype‐phenotype correlations revealed the functional consequences of the identified SNPs.

## 2 MATERIAL AND METHODS

The experimental design is summarized in Figure S1. Variants were detected using biospecimens from human subjects, and in vitro and in silico analyses determined the functional relevance of these detected variants. Additional information can be found in the Supplementary Data.

### 2.1 IBS patients and controls

SNP analysis was carried out on IBS patient and control DNA from IBS expert centres, comprising twelve cohorts from eight countries including Chile, Germany, Greece, Ireland, Spain, Sweden, the United Kingdom and the United States (Table S1). Comparative *SLC6A4* expression analyses were performed on small and large intestine biopsies from three case‐control cohorts from Spain (Barcelona) and Germany (Berlin and Erlangen‐Nürnberg) (Table S2).

All participants were Caucasian. Written informed consent was obtained from all subjects, and experiments were performed in accordance with the principles of the WMA Declaration of Helsinki and the Department of Health and Human Services Belmont Report. All studies were approved by the following local ethics committees: Germany, Heidelberg: Ethical Committee, Medical Faculty of the Heidelberg University Hospital (S067/2010); Germany, Berlin: Ethical Committee Charité Berlin Campus Mitte (No Si 285); Germany, Erlangen, Ethical Committee, Medical Faculty of the University of Erlangen‐Nürnberg (Ethik No. 4581); Chile, Santiago: Ethical Committee Hospital Clínico Universidad de Chile (Acta‐No 25/2015, No 27/2019); Greece, Athens: Ethical committee of the Aretaieio University Hospital, Medical School, National and Kapodistrian University of Athens (008/21‐11‐2017); Ireland, Cork: Clinical Research Ethics Committee (APC024); Spain, Barcelona: Ethics Committee of Hospital Universitari Vall d´Hebron (PR(AG)159/2011) and Ethics Committee of Par de Salut Mar (2005/2106/I); Sweden, Stockholm: Karolinska Institutet's Ethics Review Board (dnr 2009/1059‐31/3); Sweden, Gothenburg: Regional Ethical Review Board in Gothenburg (S489‐02 and 731‐09); United Kingdom, Manchester: NHS National Research Ethics Service, South Manchester Research Ethics Committee, Genetics of Functional GI Disorders (09/H1003/1); United Kingdom, Nottingham: clinical trial clinicaltrials.gov (identifier NCT00745004), approved by Nottingham Research Ethics Committee 2 (REC reference number 08/H0408/134)^26^; USA, St. Louis, WA: Washington University in St. Louis, Human Research Protection Office (IRB ID #: 201 103 220); USA, Los Angeles, CA: University of California Los Angeles, HORPP Office of the human research protection programme (IRB#12‐001802‐CR‐00004). Furthermore, a cohort of IBS patients and controls from the United Kingdom, United States and Canada were included in this study; these were kindly provided by Glaxo Smith Kline (UK, GenIBS/2005/0000/01).

### 2.2 Preparation of genomic DNA

Genomic DNA was prepared from blood or saliva samples taken from patients and healthy controls as described previously.^27^, ^28^

### 2.3 Genotyping of the tag SNP rs2020938

Unless otherwise stated in the Supplementary Methods section, SNP genotyping of the tagSNP rs2020938 in patients and controls from the IBS expert centres was carried out using the KASPar® assay (KBiosciences, Ltd., Hoddesdon, United Kingdom) according to the manufacturer's instructions (for primer sequences, Table S4). Thermal cycling was performed in a Mastercycler *vapo.protect* thermal cycler (Eppendorf). An initial 15 minutes incubation at 95℃ was followed by 20 cycles consisting of 10 seconds at 94℃, 5 seconds at 57℃, and 10 seconds at 72℃, followed by 23 cycles consisting of 10 s at 94℃, 5 seconds at 57℃, and 10 seconds at 72℃. After thermal cycling, results were analysed using the fluorescence plate reader of the 7500 Fast Real‐Time PCR System (Applied Biosystems). Genotyping was repeated in 10% of the samples as a quality control measure, and the same results were obtained.

### 2.4 Statistical analysis

#### 2.4.1 Statistical analysis of the genotyping data

Genotype frequencies, association analyses and tests for deviation from the Hardy‐Weinberg Equilibrium (HWE) were compared as described previously.^27^ Genotype relative risks of IBS and IBS subtypes were quantified by odds ratios (ORs) with the corresponding 95% confidence intervals (CIs) based on a logistic regression model under a dominant inheritance model (TT compared with TC and CC genotypes).^29^

#### 2.4.2 Statistical analysis for genotype‐phenotype associations in German IBS‐Net samples

Groups were compared (genotype status of *SLC6A4* SNPs) according to the following clinical phenotype features: IBS subtype (using the ROME III criteria); stool consistency (using the Bristol stool scale [BSS]) and stool frequency and IBS symptom severity (using the IBS symptom severity scale) in 134 female IBS patients from German IBS‐Net samples. Chi‐square tests were used to analyse differences in frequencies of IBS subtype (IBS‐C, IBS‐D, IBS‐M and IBS‐U) and stool consistency (hard, soft, normal). Additionally, non‐parametric Kruskal‐Wallis tests were conducted to analyse potential differences in stool frequency and IBS symptom severity. *P*‐values <.05 were considered statistically significant; a *P*‐value <.10 was considered a trend. Analyses were carried out using IBM SPSS Statistics for Windows, version 25.

### 2.5 Meta‐analysis

Results from single studies were combined using fixed and random effects meta‐analyses. Results were represented by Forest plots as follows: CIs on the OR for each study were indicated by horizontal lines, study‐specific ORs by squares proportional to the study size and combined summary estimates by a diamond with horizontal limits indicating the confidence limits. Data were analysed using the *rmeta* package from the free Software Environment for Statistical Computing R.

## 3 RESULTS

### 3.1 Seven common variants identified in the *SLC6A4* P2 promoter in a discovery cohort from the United Kingdom

The alternative promoter P2 of *SLC6A4* primarily drives expression of the serotonin transporter in the GI tract.^14^ To see whether genetic variants within this alternative promoter might regulate expression and thus contribute to or protect from IBS, we performed Sanger sequencing of this genomic region in a discovery cohort from the United Kingdom consisting of 197 IBS patients (98 IBS‐D, 99 IBS‐C) and 90 healthy control individuals. In this initial analysis, a 1,319‐bp spanning region of the P2 promoter and the downstream 5'UTR including exons 1c and 1b were screened for genetic variants (Figure 1).

Sequence data were compared with the *SLC6A4* reference sequence NC_000017 (http://www.ncbi.nlm.nih.gov/nuccore/224589808) to assess genetic variability in the P2 promoter. Twelve sequence variants were identified, seven of which were common SNPs with a minor allele frequency (MAF) above 0.05 (Table S8).

### 3.2 Identified variants are associated with IBS‐C in the discovery cohort of female patients

Association analysis, assuming a dominant model, revealed IBS‐C to be nominally associated with five of the seven common SNPs tested (Table 1).

**TABLE 1:** SNP association data of IBS‐C patients from the discovery cohort compared with healthy volunteers, both from the United Kingdom

SNP	OR	CI [lower; upper]	*P* (dominant model)
rs12150214	0.41	0.21;0.8	.008
rs2020936	0.39	0.2;0.77	.006
rs2020937	0.91	0.47;1.76	.788
rs2029038	0.44	0.23;0.85	.014
rs2020939	1.34	0.7;2.54	.373
rs25528	0.44	0.23;0.87	.017
rs6354	0.46	0.24;0.9	.023

Note

*P*‐values for the dominant model (TT compared with TC and CC genotypes). *P‐*values <.001 were rated significant after Bonferroni correction. *P*‐values smaller than 0.05 were considered ‘nominal associations’ a priori. OR, odds ratio; CI, confidence interval.

Further analysis applying the dominant model revealed a nominal association between IBS‐C and the five associated SNPs in female patients. However, due to the small sample size for males an association cannot be excluded (Table 2).

**TABLE 2:** SNP association data of common SNPs (MAF > 0.05) separated by sex in IBS‐C patients from the discovery cohort compared with female/male healthy volunteers, both from the United Kingdom

SNP	OR	CI [lower; upper]	*P* (dominant model)
Females			
rs12150214	0.41	0.20;0.83	.012
rs2020936	0.39	0.19;0.78	.008
rs2020937	1.14	0.57;2.27	.715
rs2029038	0.39	0.20;0.78	.007
rs2020939	1.14	0.58;2.24	.705
rs25528	0.44	0.22;0.89	.021
rs6354	0.47	0.23;0.95	.036
Males			
rs12150214	0.42	0.04;4.18	.428
rs2020936	0.42	0.04;4.18	.428
rs2020937	0.15	0.01;1.50	.071
rs2029038	1.27	0.18;8.79	.808
rs2020939	NA	0.00;NA	.1528
rs25528	0.48	0.05;4.81	.508
rs6354	0.37	0.04;3.65	.357

Note

*P‐*values of the dominant model. *P* < .001 was rated significant after Bonferroni correction. *P* < .05 was rated nominally a priori. OR, odds ratio; CI, confidence interval.

No deviation from HWE was detected in any groups other than IBS‐C and female IBS‐C (Table S9).

### 3.3 Haplotype block analysis identified a tag SNP for follow‐up analyses in female IBS‐C patients

**FIGURE 2:**
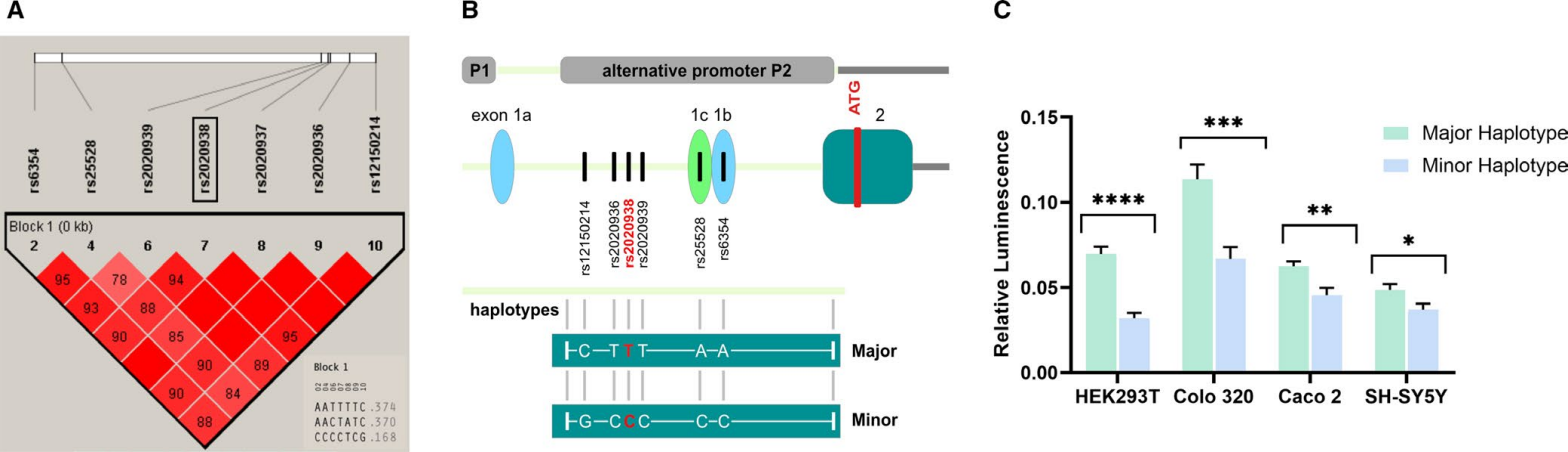
A, Genomic structure and linkage disequilibrium (LD) of the SNP markers in the *SLC6A4* P2 promoter region in female patients with IBS‐C. Strong LD is indicated by bright red (LOD ≥2, D' = 1), and no LD is indicated by white (LOD < 2, D' < 1). Pink (LOD = 2, D' < 1) and blue (LOD < 2, D' = 1) indicate intermediate LD. Association analyses of block 1 for IBS patients versus controls revealed five significant *P*‐values. All SNPs with a MAF > 5% were included. The analysed genomic region is shown as a white bar above. The relative positions of SNPs within this region are represented by vertical lines. The graphics underneath show the degree of LD among the SNPs. Red boxes without numbering represent a LD value of 100 (source: Haploview; Copyright (c) 2003‐2006 Broad Institute of MIT and Harvard). The tag SNP rs2020938 was selected for subsequent replication analysis. B, Schematic of the cloned upstream region of *SLC6A4* including the promoter regions P1 and P2, which harbour the major and minor haplotypes. The scheme is not drawn to scale. Boxes indicate exons, and lines indicate introns. ATG indicates the start codon. The upstream region of the ATG is indicated in light green, the downstream region in grey. Exons 1a, 1c, 1b, and 2 and the P1 and P2 promoters are illustrated. C, Data of the gene reporter luciferase assay. The promoter activities of the alternative *SLC6A4* promoter P2, composed of the major or the minor haplotype and harbouring the tag SNP rs2020938, were analysed in HEK293T, Colo 320, Caco 2 and SH‐SY5Y cells. Relative luciferase activities are indicated (mean ± SE). Firefly luciferase values were normalized relative to renilla luciferase values; the number of performed experiments (n = 9‐15; * *P* < .05, ** *P* < .01, *** *P* < .001, **** *P* < .0001) was corrected for multiple testing using the Benjamini, Krieger and Yekutieli procedure

Haplotype analysis of the seven common SNPs in female IBS‐C patients and female controls was performed using Haploview v4.2 (https://www.broadinstitute.org/haploview/haploview). All SNPs displayed a high linkage disequilibrium (LD), defining one haplotype block with three common haplotypes (Figure 2A). The SNP rs2020938 was selected as a representative tag SNP for subsequent analyses because it showed the strongest association with IBS‐C in female patients (*P* = .007; OR, 0.39; CI, 0.2‐0.78). To determine the functional relevance of these SNPs, we further analysed the two most contrary blocks, AATTTC and CCCCTCG, hereafter termed major and minor, respectively.

### 3.4 Functional readout of SNPs in luciferase reporter assays

To investigate the functional relevance of the SNPs arranged in the two haplotype blocks, representative genomic regions were amplified from the genome of respective carriers, cloned upstream of a luciferase reporter cassette and sequence verified (Figure 2B). The major and minor haplotype reporter constructs were analysed in four human cell lines: HEK293T embryonic kidney cells, SH‐SY5Y neuroblastoma cells, as well as Caco 2 and Colo 320, both colon carcinoma cells (Figure 2C). All cell lines endogenously express *SLC6A4* as shown by RT‐PCR (see Figure S2). Luciferase reporter assays revealed that the minor haplotype in the P2 promoter region carrying the rs2020938 variant drives less gene expression than the major haplotype does in all four cell lines (Figure 2C).

### 3.5 Replication analysis of rs2020938 in twelve additional cohorts from eight countries confirmed the association with IBS‐C in female patients

To validate the association between rs2020938 and IBS‐C in female patients from the UK discovery sample, we genotyped rs2020938 in 1978 additional IBS patients and 6,038 controls from twelve cohorts in eight countries: two from the United Kingdom, two from Germany, three from the United States and one each from Ireland, Sweden, Spain, Greece and Chile (Table S3). Cohorts with 30 or fewer individuals in subgroup analyses were not included in the meta‐analysis. The average number of IBS patients per study was 167 (range, 34‐455).

**FIGURE 3:**
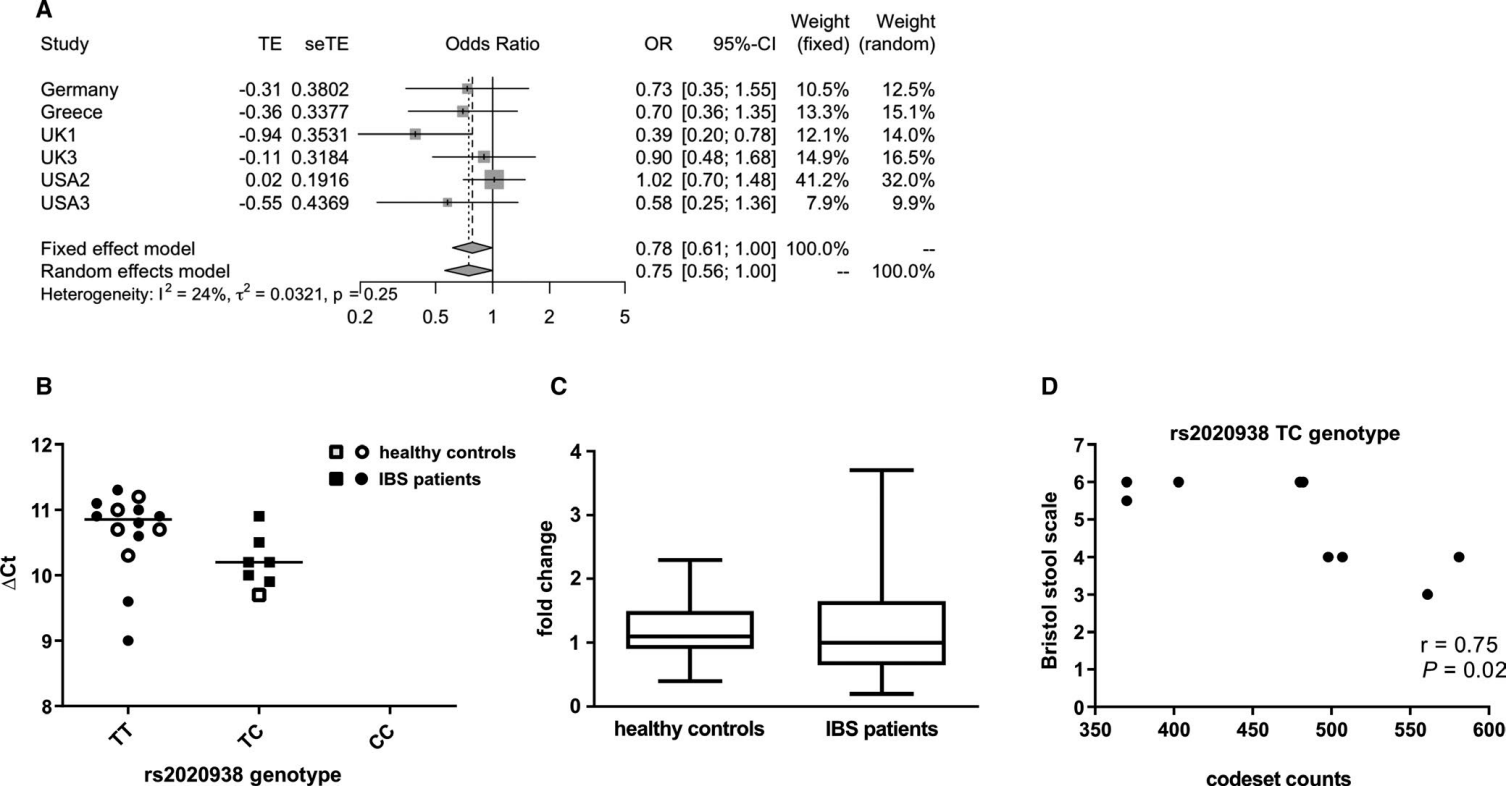
A, Forest plots illustrating genotype risks of rs2020938 of IBS‐C in females. Genotype risk is shown as odds ratios (OR) with corresponding 95% confidence intervals (CIs) and is based on a logistic regression model under dominant genetic penetrance. CIs for each study are indicated by horizontal lines, ORs by squares that reflect study sizes, and summary estimates by diamonds with horizontal limits at confidence limits and with a width inversely proportional to the standard error. Only data sets with more than 30 individuals per genotype group were included. The I^2^ of 24% and τ^2^ of 0.03 suggest relatively low heterogeneity between cohorts. Both fixed effects and random effects models result in similar effect estimates (fixed effect model: OR, 0.782; CI, 0.614‐0.995; random effects model: OR, 0.747; CI, 0.559‐1.000; *P*‐values = .0458 and .05, respectively). B‐D, Expression data of the *SLC6A4* P2‐driven isoform from tissue samples from the jejunum: B, qPCR results showing jejunal mucosal expression of *SLC6A4* P2 isoform and genotype correlation of the tag SNP rs2020938 representative for the polymorphic P2 region. C, Comparative expression analysis of IBS and healthy controls by qPCR. D, Correlation analysis of nCounter expression data in TC carriers of rs2020938

Meta‐analysis of genotype data from 2,175 IBS patients and 6,128 controls confirmed the association between rs2020938 and IBS‐C in female patients (OR = 0.75 and 0.78, *P =* .0458 and .05 using a fixed and random effects model) (Figure 3A). Analyses involving male patients or non‐IBS‐C subtypes did not show a statistically significant association (data not shown).

In all control individuals and most IBS patients, no deviations from HWE expectations were detected except for IBS‐C and female IBS‐C patients from the United Kingdom (*P* = .027‐0.041) and IBS overall, IBS‐C and female IBS‐C patients from Greece (*P* = 1.65 × 10^‐12^‐1.68 × 10^‐12^) (Table S9).

### 3.6 Comparative expression analysis of *SLC6A4* in different GI regions

**FIGURE 4:**
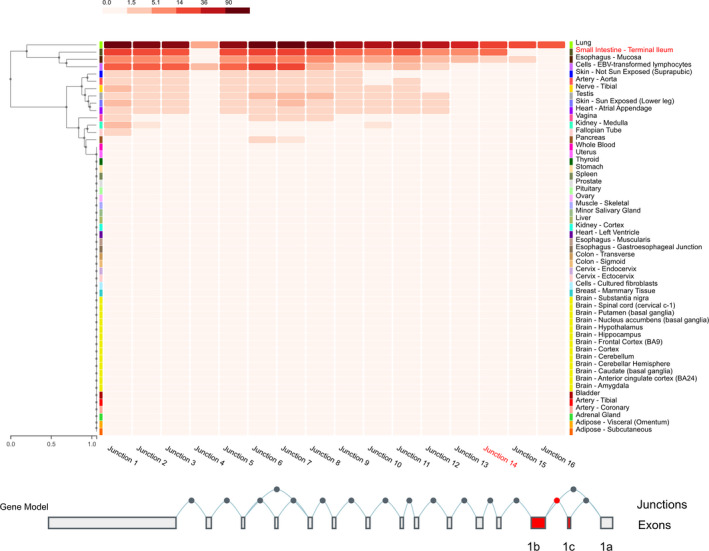
Exon‐exon junction expression data from different tissues modified from the GTEx portal (v8). The P1‐driven isoforms are represented by junction 15 and 16, whereas the P2‐driven isoform is represented by junction 14 (marked in red), which shows a highly prominent expression compared with 15 and 16 in the small intestine (terminal ileum, data from n = 187 donors included, median TPM 4.219). All other tissues, except the lung, show much lower expression levels (eg oesophagus n = 555 donors, median TPM 1.645, all others: median TPM below 0.2). TPM, transcripts per million (adapted from Junction Expression of *SLC6A4*: ENSG00000108576.9 solute carrier family 6 member 4 [Source: HGNC Symbol; Acc:HGNC:11050] GTEx portal: https://gtexportal.org/home/)

Analysis of expression driven by the P1 and P2 promoter in different intestinal regions confirmed that the P2 promoter primarily drives *SLC6A4* expression within the small intestine. In particular, robust expression was detected in the jejunum (Ct: 27‐31) and even more pronounced in the ileum (Ct: 22‐27). In contrast, within the large intestine Ct values were much higher, within colon (Ct: 30‐37) and the sigma (Ct 31‐37) and thus, the P2‐driven isoform not found to be consistently expressed. Of note, the P1 promoter‐driven transcript was neither detectable at a robust level in the small nor in the large intestine (Table S10). This is in line with expression data of different tissues from the GTEx portal (Release v08, https://gtexportal.org/home/), which shows highest expression in the lung, followed by the small intestine (terminal ileum, data from n = 187 donors, median TPM (transcripts per million) = 4.219). Expression in other tissues was much lower (eg oesophagus, n = 555 donors, median TPM = 1.645, all others: median TPM below 0.2). In addition, isoform‐specific expression analysis confirmed that the P2‐driven isoform is the most abundantly expressed isoform in the small intestine (Figure 4).

Further follow‐up studies in tissue samples of IBS patients and controls revealed that the homozygous genotype TT of the major allele of rs2020938 correlated with significantly altered expression in the jejunum (*P* = .0398), corroborating a functional impact of the SNP on *SERT* expression (Figure 3B). In contrast, overall expression was not different between IBS patients and control individuals (Figure 3C). Moreover, ileum samples from a German centre showed no correlation with the rs2020938 genotype, potentially because of the small number of variant carriers (data not shown).

### 3.7 Genotype‐phenotype correlation

Correlation analysis of nCounter expression data of jejunum samples was performed separately in IBS patients of TT or TC genotype carriers from the Spanish centre. This revealed a significant correlation between *SLC6A4* expression and stool consistency (assessed by the BSS) in TC cases only (*r* = .75; *P* = .02) (Figure 3D). Hence, lower *SLC6A4* expression in TC carriers is associated with softer stools.

These findings were corroborated by genotype‐phenotype association data (IBS subtype, stool consistency, stool frequency and IBS symptom severity) of 134 female IBS patients from the IBS‐Net in Germany. No associations were found for IBS subtype, stool frequency or symptom severity. However, *SLC6A4* TC/CC carriers showed a trend for softer stools (according to the BSS) compared with TT carriers (*P* = .053).

### 3.8 Impact of DNA variants on transcription factor binding capacity

**FIGURE 5:**
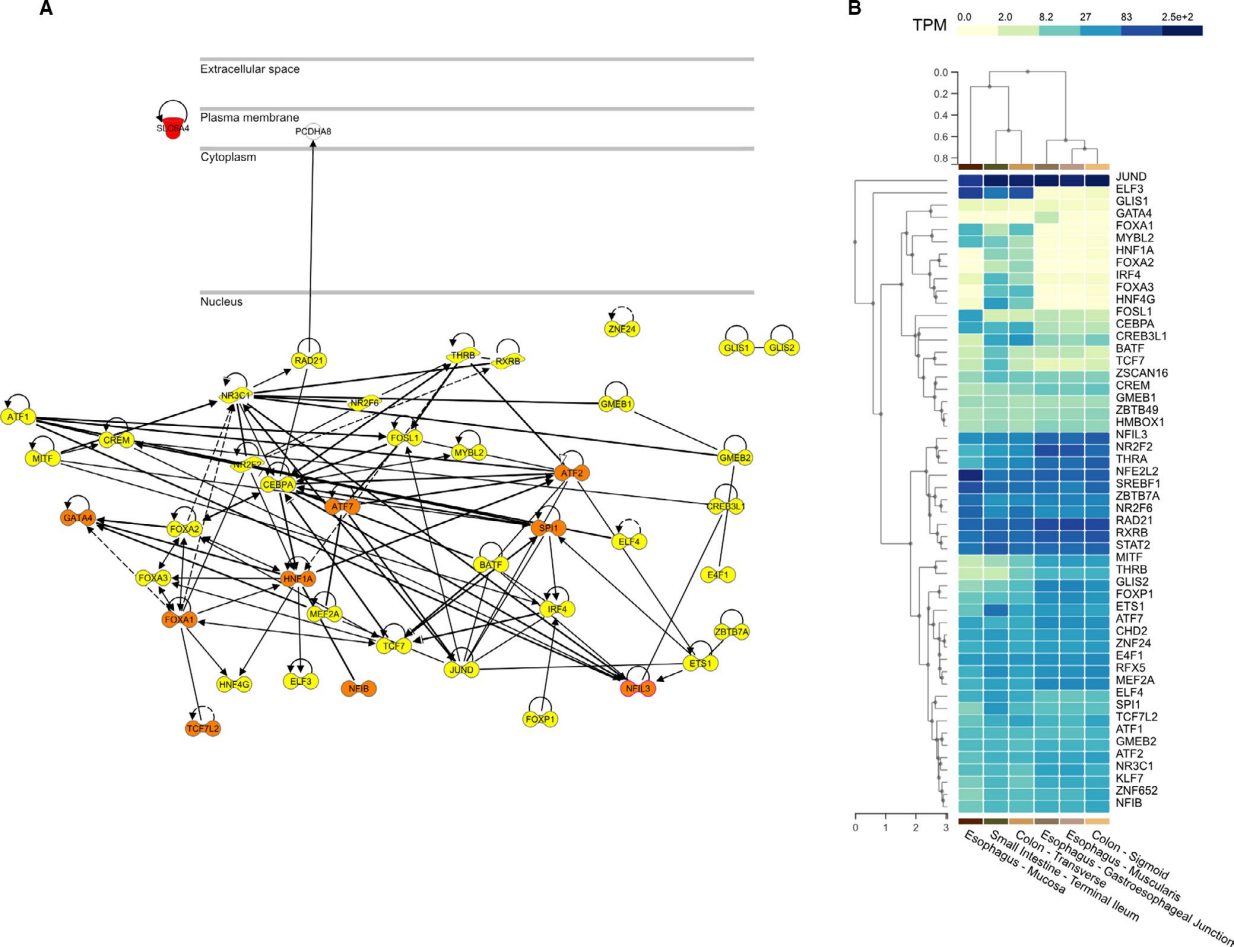
A, Network analysis B, GTEx expression data of the 52 transcription factors (highlighted in yellow) predicted to bind differently within the polymorphic P2 promoter region. The factors coloured in orange have previously been implicated in GI disorders

We performed comparative transcription factor binding site analysis in the major and the minor (protective) haplotype genomic regions where the tag SNP rs2020938 resides. We used the ePOSSUM2 online tool (https://www.mutationdistiller.org/ePOSSUM2/) to predict the impact of DNA variants on transcription factor binding. This revealed 52 novel binding sites in the variant sequence, 32 of which were predicted by more than three models (Table S11). Ingenuity pathway analysis assembled these genes into three major networks according to the top diseases and functions as follows: 1. gene expression, cell cycle and cellular development; 2. gene expression, cell morphology and humoral immune response; and 3. gene expression, auditory disease and inflammatory disease. Nine factors assembled in the overlay network have been implicated in GI disorders (Figure 5A, highlighted in orange). Data reported in the GTEx portal (release v08, https://gtexportal.org/home/) confirmed that most of these genes are moderately to highly expressed in GI tissues (Figure 5B). Subsequent analysis of ChIP Atlas data (https://chip‐atlas.org/search) to collect further evidence and validate ePOSSUM data revealed all predicted transcription factors but nine to be included. Interestingly, eight of the predicted transcription factors were found to indeed co‐precipitate and thus interact with *SLC6A4*: Among those, ETS1, FOXP1, MITF, RAD21 and ZBTB7A are also included in the interaction network (Figure 5, Table S11).

## 4 DISCUSSION

We have discovered a novel, functionally relevant cis‐regulatory promoter haplotype in the *SLC6A4* gene that is associated with IBS‐C in females. This further supports the physiological relevance of SERT in IBS pathogenesis and consolidates the importance of the serotonergic pathway in regulating gut homeostasis and IBS development.

It remains unclear whether the deletion/insertion polymorphism s/l in the P1 promoter region of the *SLC6A4* gene plays a role in IBS development.^11^ This prompted us to investigate the alternative promoter, P2. The s/l polymorphism has been associated with increased susceptibility to stress‐provoked psychopathologies^20^, ^30^ that are comorbid with IBS. We wondered whether the gut‐predominant isoform might help to dissect the role of SERT in the GI tract.

A recent comprehensive meta‐analysis based on more than 7000 individuals, including more than 3400 IBS and 3600 control cases, reported that the *SLC6A4* s/l polymorphism correlated with the risk of IBS‐C in Asians and Caucasians.^31^ Here, we show that the *SLC6A4* P2 promoter drives expression in the gut mucosa and, predominantly, in the small intestine. Our finding that the P2‐driven isoform is predominantly expressed in the small intestine is supported by data in the GTEx portal (https://gtexportal.org/home/). Of note, the predominant expression of SERT within the small intestine is also in line with previous studies.^32^, ^33^ The lack of robust expression of the *SLC6A4* P1‐driven isoform in the small and large intestine challenges the role of the P1 s/l polymorphism in the gut. Furthermore, how the P1 promoter‐driven isoform could shape a GI phenotype is unknown. The reported association of the P1 promoter s/l polymorphism with IBS‐C may be attributed to comorbid conditions, such as behavioural phenotypes. This is supported by the fact that 80% of IBS patients present with some form of psychological comorbidity.^2^ However, the authors of the meta‐analysis did not stratify for comorbid phenotypes like anxiety and depression, so this remains elusive at present.

How factors such as stress and trauma influence gene‐environment and gene‐gene interactions has also not been addressed to date.^31^ Sex differences were recently described in the modulation of serotonin in the thalamus, and 5‐HT‐binding studies showed that 5‐HTTP may affect males and females differently. Furthermore, serotonergic signalling is affected differently in males and females with affective disorders, suggesting that sex‐dependent availability of serotonin transporters in the thalamus contributes to the risk of affective disorders.^24^, ^34^, ^35^ Recent reports of an association between rare *SLC6A4* variants and psychiatric comorbidity in IBS are very much in line with the biopsychological model of IBS.^36^

Variants in the *SLC6A4* promoter can affect the response to treatment. For example, IBS‐D patients with the P1 l/l genotype respond better to the 5‐HT_3_ receptor antagonist alosetron,^37,37^ whereas IBS‐C patients with the P1 polymorphism l/l respond poorly to the 5‐HT_4_ receptor agonist tegaserod compared with s/l or s/s carriers.^38^ Therefore, our finding that the SNP rs2020938 is associated with IBS‐C in females may be relevant to novel pharmacogenetic treatments.

In vitro luciferase assays supported our initial hypothesis that the protective SNP rs2020938 in P2 reduces expression of the GI‐predominant SERT isoform. Follow‐up analysis of tissue samples from IBS patients and controls showed that carriers of the rs2020938 minor allele seem to have higher SERT expression in the jejunum; this may confirm a functional impact of this SNP on SERT expression. Further genotype‐phenotype correlations in gut tissue of IBS patients seem to corroborate our findings—carriers of the protective C allele with lower expression of the serotonin transporter P2 promoter‐driven isoform had softer stools. This is in line with the trend for an association between TC/CC carrier status and softer stools observed within the German IBS‐Net replication cohort. Our genotype‐phenotype correlations also agree with earlier studies showing that 5‐HT levels correlate negatively with SERT expression^15^, ^22^, ^23^ in IBS‐D, which is characterized by softer stools. Decreased levels of 5‐HT have also been reported in IBS‐C^22^, ^23^, ^24^ and might be caused by increased SERT expression. This intriguing finding shows, for the first time, that bowel habits correlate with the carrier status of polymorphisms within the *SLC6A4* P2 promoter, which drives expression in the GI tract.

The potential functional impact of rs2020938 is emphasized by recent data in the OMNI database (https://omni.telentilab.com/search=rs2020938/page=1) indicating that rs2020938 presumably acts as a modifier. Moreover, single‐tissue eQTLs analysis within the GTEx portal showed significant eQTL signatures for rs2020938 in brain regions such as the amygdala and the cerebellum for a transcript termed AC006050.2 and for *SUZ12P* (SUZ12 polycomb repressive complex 2 subunit pseudogene) and *SSH2* (slingshot protein phosphatase 2) in the tibial nerve, but in no other nerve or gut tissue. (Table S12). AC006050.2 and for *SUZ12P* have no annotated function. AC006050.2 seems to be a non‐coding RNA, and *SUZ12P* is a pseudogene. *SSH2* belongs to the SSH phosphatase gene family composed of three members (*SSH1, SSH2* and *SSH3*) known to control essential cellular functions, including invasion, migration and motility. *SSH2* was recently reported to drive proliferation in colon cancer stem cells.^39^ How these eQTL findings might relate to IBS remains unclear.

The polymorphic P2 promoter region harbours more than 50 novel transcription factor binding sites. These transcription factors have been annotated to three major networks, including gene expression and cellular development, morphology and function, and humoral immune response and inflammatory disease. Furthermore, the GTEx database shows that most of these transcription factors are robustly expressed in GI tissues, indicating a possible relevance to the pathogenesis of IBS. Interestingly, nine have been implicated in GI disorders; for example, *Nfil3* deficiency has been associated with colitis, *NFIL3* is a susceptibility gene for inflammatory bowel disease,^40^, ^41^ and *TCF7L2* has been implicated in colorectal cancer.^42^, ^43^

GO enrichment analysis recently revealed serotonin pathway genes, particularly those involved in serotonin re‐uptake, to be the most differentially expressed genes between IBS patients and healthy controls.^44^ Furthermore, interaction of eight of these predicted transcription factors was proven based on ChIP Atlas data.

Regulation of expression by alternative promoters and various splicing isoforms leads to profound molecular heterogeneity. Within GenBank, seven human *SLC6A4* isoforms driven by the P1 and P2 promoters are currently annotated (AK313166 and AK308014 by P1 and L05568, AK309538, AY902473, BC069484 and X70697 by P2) underlining the molecular heterogeneity of *SLC6A4*. Furthermore, gene expression is regulated by micro‐RNAs (miRNAs) in IBS.^11^ Of note, the miR‐16 family that we recently found to be downregulated in IBS^45^, ^46^ was reported to modulate SERT expression within the brain^47^ and presumably also regulates SERT expression in the GI tract. Furthermore, miR‐24 was found to be upregulated, whereas SERT was downregulated in the intestinal mucosa of IBS‐D patients.^48^ In an animal model of IBS, miR‐24 was found to trigger symptoms including visceral pain.^48^ *SLC6A4* regulation was recently summarized in a comprehensive review.^49^ Regulation of *SLC6A4* expression is complex, and it is currently unknown how the different isoforms are expressed and how miRNAs and/or polymorphic sites modulate the various functions of the serotonergic system. Bidirectional communication between the gut and the brain via the brain‐gut/gut‐brain axis might be modified by cis‐regulatory polymorphisms that drive gene expression and control gene function in specific subregions/organs differently.

Our study has some limitations. We combined data from 13 centres from different countries, so methodological discrepancies exist. Only one SNP was genotyped, so we could not correct our results for population stratification using genetic principal components. To mitigate potential bias, we only included individuals of European ancestry. Since all participants were Caucasians, our association finding may not equally apply to the other populations. Also, the SNP genotype frequencies significantly deviated from the HWE expectation in patients from the Greek cohort, which we cannot explain at present. However, the estimated effect size and direction are in line with the overall results.

Another limitation is that we did not define the IBS phenotype according to a uniform symptom classification—we used Rome II and/or Rome III criteria. The Rome III criteria^50^ allow a wider variation in diagnostic identification of IBS, particularly of the IBS subtypes. We also analysed more females than males because the prevalence of IBS is higher in women. Accordingly, we cannot conclude that the identified SNP would not be associated with IBS‐C in males in a cohort containing more male patients.

GI phenotypes were not examined in control individuals, so we cannot exclude that IBS patients were in the control sample. Moreover, because of symptom‐based classifications and heterogeneity, we cannot exclude that enrolled patients had the same diseases and aetiopathogenetic mechanisms. For genetic studies, a purely symptom‐based IBS classification (based solely on bowel function) is not specific enough to identify mechanistically diverse phenotypes of IBS or its subgroups.^11^ Therefore, additional parameters, as well as intermediate phenotypes or quantitative traits, are mandatory to dissect genetic patterns underlying IBS and to correlate these to symptoms/markers. As recently established by GENIEUR,^46^ deep phenotypic characterization of patients is mandatory and should be based on the following criteria: clinical examination and specific questionnaires (assessing not only GI but also psychiatric comorbidity, personality traits and somatization), assessment of laboratory parameters and tissue sampling to follow‐up changes in expression in certain candidates. Control individuals should be characterized in a similar way to avoid including IBS patients in the control sample. However, most of the samples analysed in this study were collected before the GENIEUR phenotyping tool was established, so do not follow that standard.^51^ Some studies showed that the IBS subtype may change over time, which is another limitation. For example, one study found that IBS‐C changed to IBS‐D or *vice versa* in 14% of female patients.^52^ In addition, many of our samples came from tertiary referral centres, so may not be generalizable to all IBS patients.

Another limitation is the small number of samples in the differential expression analysis. However, a strength of our study is that we analysed expression in the small and large intestine, unlike previous studies that focussed on only one or two gut regions.^53^

In conclusion, we confirmed that the novel promoter P2 is the predominant driver of SERT expression in the gut and that a functionally relevant SNP in *SLC6A4* is associated with IBS‐C in females. This underlines the relevance of SERT in IBS pathogenesis. To what extent this SNP shapes the GI phenotype in IBS and how it interferes with other brain‐related genetic variants and impacts bidirectional communication between the gut and the brain remain unanswered. Our future studies within the international H2020 consortium DISCOvERIE (*Development,* *dIagnosis and prevention of gender‐related Somatic and mental COmorbiditiEs in iRritable* *Bowel Syndrome In Europe*, www.DISCOvERIE.eu) that implemented GENIEUR guidelines will allow us to investigate how these variants correlate with behaviour, pain perception and bowel habits and how far gene‐gene and gene‐environment interactions affect individual susceptibility to chronic GI disorders. A better understanding of potential SERT regulators is of clinical importance and may provide insight into IBS pathophysiology and SERT‐directed therapeutic interventions.

## CONFLICT OF INTEREST

The authors declare there is no conflict of interest.

## AUTHOR CONTRIBUTIONS

**Sandra Mohr:** Formal analysis (lead); Investigation (lead); Methodology (lead); Writing‐review & editing (supporting). **Nikola Fritz:** Formal analysis (equal); Investigation (equal); Methodology (equal); Writing‐review & editing (supporting). **Christian Hammer:** Data curation (supporting); Formal analysis (supporting); Methodology (supporting); Writing‐review & editing (supporting). **Cristina** **Martínez:** Formal analysis (supporting); Investigation (supporting); Methodology (supporting); Supervision (supporting); Writing‐review & editing (supporting). **Sabrina** **Berens:** Data curation (supporting); Formal analysis (supporting); Writing‐review & editing (supporting). **Stefanie** **Schmitteckert:** Formal analysis (supporting); Investigation (supporting); Supervision (supporting); Writing‐review & editing (supporting). **Verena Wahl:** Formal analysis (supporting); Investigation (supporting); Writing‐review & editing (supporting). **Malin Schmidt:** Formal analysis (supporting); Investigation (supporting); Writing‐review & editing (supporting). **Lesley A. Houghton:** Formal analysis (supporting); Investigation (supporting); Resources (supporting); Writing‐review & editing (supporting). **Miriam Goebel‐Stengel:** Formal analysis (supporting); Investigation (supporting); Resources (supporting); Writing‐review & editing (supporting). **Maria** **Kabisch:** Data curation (supporting); Formal analysis (supporting); Writing‐review & editing (supporting). **Dorothea Goetz:** Data curation (supporting); Formal analysis (supporting); Writing‐review & editing (supporting). **Irina** **Milovač:** Formal analysis (supporting); Investigation (supporting); Writing‐review & editing (supporting). **Mauro** **d'Amato:** Data curation (supporting); Formal analysis (supporting); Resources (supporting); Writing‐review & editing (supporting). **Tenghao Zheng:** Data curation (supporting); Formal analysis (supporting); Writing‐review & editing (supporting). **Ralph** **Röth:** Formal analysis (supporting); Investigation (supporting); Methodology (supporting); Writing‐review & editing (supporting). **Hubert** **Mönnikes:** Resources (supporting); Writing‐review & editing (supporting). **Felicitas Engel:** Resources (supporting); Writing‐review & editing (supporting). **Annika Gauss:** Resources (supporting); Writing‐review & editing (supporting). **Jonas** **Tesarz:** Formal analysis (supporting); Resources (supporting); Writing‐review & editing (supporting). **Martin** **Raithel:** Formal analysis (supporting); Resources (supporting); Writing‐review & editing (supporting). **Viola Andresen:** Resources (supporting); Writing‐review & editing (supporting). **Thomas** **Frieling:** Resources (supporting); Writing‐review & editing (supporting). **Jutta Keller:** Resources (supporting); Writing‐review & editing (supporting). **Christian** **Pehl:** Resources (supporting); Writing‐review & editing (supporting). **Christoph Stein‐Thoeringer:** Resources (supporting); Writing‐review & editing (supporting). **Gerald Clarke:** Resources (supporting); Writing‐review & editing (supporting). **Paul J. Kennedy:** Data curation (supporting); Resources (supporting); Writing‐review & editing (supporting). **John F**. **Cryan:** Resources (supporting); Writing‐review & editing (supporting). **Timothy G**. **Dinan:** Resources (supporting); Writing‐review & editing (supporting). **Eamonn M. Quigley:** Resources (supporting); Writing‐review & editing (supporting). **Robin Spiller:** Data curation (supporting); Resources (supporting); Writing‐review & editing (supporting). **Carroll** **Beltrán:** Data curation (supporting); Formal analysis (supporting); Resources (supporting); Writing‐review & editing (supporting). **Ana Maria Madrid:** Data curation (supporting); Resources (supporting); Writing‐review & editing (supporting). **Verónica Torres:** Data curation (supporting); Resources (supporting); Writing‐review & editing (supporting). **Edith Pérez de Arce:** Data curation (supporting); Resources (supporting); Writing‐review & editing (supporting). **Wolfgang Herzog:** Resources (supporting); Writing‐review & editing (supporting). **Emeran E. Mayer:** Resources (supporting); Writing‐review & editing (supporting). **Gregory** **Sayuk:** Data curation (supporting); Resources (supporting); Writing‐review & editing (supporting). **Maria** **Gazouli:** Data curation (supporting); Formal analysis (supporting); Investigation (supporting); Resources (supporting); Writing‐review & editing (supporting). **George** **Karamanolis:** Data curation (supporting); Resources (supporting); Writing‐review & editing (supporting). **Lejla Kapur Pojskič:** Supervision (supporting); Writing‐review & editing (supporting). **Mariona Bustamante:** Data curation (supporting); Resources (supporting); Writing‐review & editing (supporting). **RaquelRabionet:** Data curation (supporting); Resources (supporting); Writing‐review & editing (supporting). **Xavier** **Estivill:** Resources (supporting); Writing‐review & editing (supporting). **André** **Franke:** Resources (supporting); Writing‐review & editing (supporting). **Wolfgang** **Lieb:** Resources (supporting); Writing‐review & editing (supporting). **Guy** **Boeckxstaens:** Resources (supporting); Writing‐review & editing (supporting). **Mira** **Wouters:** Resources (supporting); Writing‐review & editing (supporting). **Magnus** **Simrén:** Data curation (supporting); Resources (supporting); Writing‐review & editing (supporting). **Gudrun A**. **Rappold:** Formal analysis (supporting); Resources (supporting); Writing‐review & editing (supporting). **Maria Vicario:** Data curation (supporting); Resources (supporting); Writing‐review & editing (supporting). **Javier Santos:** Resources (supporting); Writing‐review & editing (supporting). **Rainer Michael** **Schäfer:** Data curation (supporting); Resources (supporting); Writing‐review & editing (supporting). **Justo Lorenzo Bermejo:** Conceptualization (equal); Formal analysis (equal); Methodology (equal); Supervision (equal); Writing‐review & editing (supporting). **Beate Niesler:** Conceptualization (lead); Funding acquisition (lead); Project administration (lead); Resources (lead); Supervision (lead); Writing‐original draft (lead); Writing‐review & editing (lead).

## Supporting information

Supplementary Material

## Data Availability

The data that support the findings of this study are available from the corresponding author upon reasonable request
