# Supplementary material for: The alternative serotonin transporter promoter P2 impacts gene function in females with irritable bowel syndrome
Source: J Cell Mol Med. 2021 Jun 24;25(16):8047–61. doi: 10.1111/jcmm.16736 (PMC8358858; doi:10.1111/jcmm.16736)
Supplement: Supplementary file 1 — Supplementary Material [file JCMM-25-8047-s001.docx]

#### Supplementary Data

# The alternative serotonin transporter promoter P2 impacts gene function in females with irritable bowel syndrome

Sandra Mohr^1^, Nikola Fritz^1^, Christian Hammer^2,3^, Cristina Martínez^1,4,5^, Sabrina Berens^6^_,_ Stefanie Schmitteckert^1^, Verena Wahl^1^, Malin Schmidt^1,7^, Lesley A. Houghton^8,9^, Miriam Goebel-Stengel^10,11^, Maria Kabisch^12^, Dorothea Götze^1^, Irina Milovač^13^, Mauro D’Amato^14,15,16,17^, Tenghao Zheng^14,15^, Ralph Röth^1,18^, Hubert Mönnikes^19^, Felicitas Engel^6^, Annika Gauss^20^, Jonas Tesarz^6^, Martin Raithel^21^, Viola Andresen^22^, Thomas Frieling^23^, Jutta Keller^22^, Christian Pehl^24^, Christoph Stein-Thöringer^25^, Gerard Clarke^26,27^, Paul J. Kennedy^26,27^, John F. Cryan^26,27,28^, Timothy G. Dinan^26,27^, Eamonn M. M. Quigley^27,29^, Robin Spiller^30^, Caroll Beltrán^31^, Ana María Madrid^31^, Verónica Torres^31^, Wolfgang Herzog^6^, Emeran A. Mayer^32^, Gregory Sayuk^33^, Maria Gazouli^34^, George Karamanolis^35^, Lejla Kapur-Pojskič^36^, Mariona Bustamante^37,38^, Raquel Rabionet^39^, Xavier Estivil^37^, André Franke^40^, Wolfgang Lieb^41^, Guy Boeckxstaens^42^, Mira M. Wouters^42^, Magnus Simrén^43^, Gudrun A. Rappold^1,44^, Maria Vicario^45^, Javier Santos^45^, Rainer Schaefert^46,47^, Justo Lorenzo-Bermejo^12^, Beate Niesler^1,18,44^

^1^Institute of Human Genetics, Department of Human Molecular Genetics, Heidelberg University Hospital, Heidelberg, Germany

^2^Department of Cancer Immunology, Genentech, South San Francisco, CA, USA

^3^Department of Human Genetics, Genentech, South San Francisco, CA, USA

^4^Institut de Recerca Biomèdica de Lleida (IRBLleida), Lleida, Spain

^5^Lleida Institute for Biomedical Research Dr. Pifarré Foundation (IRBLleida), Lleida, Spain

^6^Department of General Internal Medicine and Psychosomatics, Heidelberg University Hospital, Heidelberg, Germany

^7^Department of Translational Brain Research, Central Institute of Mental Health

^8^University of Leeds, St. James’s University Hospital, Leeds, UK

^9^Mayo Clinic, Jacksonville, FL, USA

^10^Department of Psychosomatic Medicine, University Hospital Tübingen, Tübingen, Germany

^11^Department of Internal Medicine and Gastroenterology, HELIOS Clinic Rottweil, Rottweil, Germany

^12^Institute of Medical Biometry and Informatics, Heidelberg University, Heidelberg, Germany

^13^Faculty of Medicine, University Banja Luka, Banja Luka, Bosnia and Herzegovina

^14^School of Biological Sciences, Monash University, Clayton VIC, Australia

^15^Unit of Clinical Epidemiology, Department of Medicine Solna, Karolinska Institutet, Stockholm, Sweden

^16^Department of Gastrointestinal and Liver Diseases, Biodonostia HRI, San Sebastián, Spain

^17^IKERBASQUE, Basque Foundation for Science, Bilbao, Spain

^18^nCounter Core Facility, Department of Human Molecular Genetics, Heidelberg UniversityHospital, Heidelberg, Germany

^19^Martin-Luther-Hospital, Berlin, Germany

^20^Department of Gastroenterology, Infectious Diseases and Intoxications, Heidelberg University, Heidelberg, Germany

^21^University of Erlangen, Erlangen, Germany

^22^Israelitisches Krankenhaus, Hamburg, Germany

^23^Helios Klinik Krefeld, Krefeld, Germany

^24^Krankenhaus Vilsbiburg, Vilsbiburg, Germany,

^25^German Cancer Research Center, Heidelberg, Germany

^26^Department of Psychiatry and Neurobehavioral Science, University College Cork, Cork, Ireland

^27^APC Microbiome Ireland, University College Cork, Cork, Ireland

^28^Department of Anatomy and Neuroscience, University College Cork, Cork, Ireland

^29^Lynda K. and David M. Underwood Center for Digestive Disorders, Houston Methodist Hospital, Weill Cornell Medical College, Houston, TX, USA

^30^Nottingham Digestive Diseases Centre, University of Nottingham, Nottingham, UK

^31^Gastroenterology Unit, Hospital Clínico Universidad de Chile, Medicine Department, Universidad de Chile, Santiago de Chile, Chile

^32^Oppenheimer Center for Neurobiology of Stress, University of California, Los Angeles, CA, USA

^33^Washington University School of Medicine, St. Louis, MO, USA

^34^Laboratory of Biology, Medical School, National and Kapodistrian University of Athens, Athens Greece

^35^Academic Department of Gastroenterology, Medical School, National and Kapodistrian University of Athens, "Laikon" General Hospital, Athens, Greece

^36^University of Sarajevo, Institute for Genetic Engineering and Biotechnology, Sarajevo, Bosnia and Herzegovina

^37^CRG, Centre for Genomic Regulation, Barcelona, Spain

^38^ISGlobal, Barcelona, Spain

^39^Department of Genetics, Microbiology and Statistics, Faculty of Biology, IBUB, Universitat de Barcelona; CIBERER, IRSJD, Barcelona, Spain

^40^Institute of Clinical Molecular Biology, Kiel, Germany

^41^Institute of Epidemiology, Kiel, Germany

^42^TARGID, University Hospital Leuven, Leuven, Belgium

^43^Institute of Medicine, University of Gothenburg, Gothenburg, Sweden

^44^Interdisciplinary Center for Neurosciences (IZN), Heidelberg University, Heidelberg, Germany

^45^Institut de Recerca Vall d’Hebron, Hospital Vall d'Hebron, Passeig de la Vall d'Hebron, Barcelona, Spain

^46^Department of Psychosomatic Medicine, Division of Internal Medicine, University Hospital Basel, Basel, Switzerland

^47^Faculty of Medicine, University of Basel, Basel, Switzerland

**SD Material and Methods**

**Tables**

**SD Table 1** Cohorts from IBS expert centers

IBS, irritable bowel syndrome; IBS-A, alternating IBS; IBS-C, constipation-predominant IBS; IBS-D, diarrhea-predominant IBS; IBS-M, mixed IBS; IBS-U, unspecified IBS; yrs, years.

| **Cohort** |  | **Female** | **Male** | **Age yrs (mean)** | **Rome II / III** |
| --- | --- | --- | --- | --- | --- |
| UK1 | IBS-D | 67 | 31 | 18-66 (41.6) | II |
|  | IBS-C | 95 | 5 | 18-65 (40.1) |  |
|  | Controls | 60 | 32 | 18-63 (35.6) |  |
| "Belgium"= USA2&UK3 | IBS-D | 323 | 121 | 18-70 (44.3) | II |
|  | IBS-C | 305 | 25 | 18-81 (43.7) |  |
|  | Controls | 506 | 158 | 18-83 (41.1) |  |
| Chile | IBS-D | 12 | 2 | 24-77 (48.4) | III |
|  | IBS-C | 15 | 1 | 22-80 (48) |  |
|  | IBS-M | 31 | 9 | 19-58 (33.7) |  |
|  | IBS-U | 1 | 0 | 54 |  |
|  | Controls | 21 | 17 | 18-69 (32.7) |  |
| Germany1 | IBS-D | 72 | 42 | 19-79 (44.7) | III |
|  | IBS-C | 40 | 8 | 16-71 (45.5) |  |
|  | Controls | 138 | 203 | n.a. |  |
| Germany2 | IBS-D | 70 | 41 | 13-79 (41.6) | III |
|  | IBS-C | 18 | 7 | 18-76 (45.7) |  |
|  | IBS-M | 75 | 47 | 14-88 (38.8) |  |
|  | IBS-U | 7 | 2 | 27-74 (49.6) |  |
|  | Controls | 510 | 651 | 19-77 (53.6) |  |
| Greece | IBS-D | 27 | 8 | 21-72 (44.2) | III |
|  | IBS-C | 91 | 23 | 17-73 (51.7) |  |
|  | IBS-M | 4 | 2 | 35-66 (56.2) |  |
|  | Controls | 74 | 69 | 19-94 (54.2) |  |
| Ireland | IBS-D | 3 | 1 | 22-32 (27.25) | III |
|  | IBS-C | 3 | 0 | 19-43 (20.3) |  |
|  | IBS-M | 25 | 4 | 19-51 (29.6) |  |
|  | IBS-U | 0 | 0 | n.a. |  |
|  | Controls | 38 | 12 | 18-50 (29.3) |  |
| Spain | IBS-D | 43 | 17 | 19-55 (36.9) | III |
|  | IBS-C | 0 | 0 | n.a. |  |
|  | IBS-M | 0 | 0 | n.a. |  |
|  | IBS-U | 0 | 0 | n.a. |  |
|  | Controls | 9 | 5 | 19-54 (25.7) |  |
| Sweden Mosaic | IBS-D | 16 | 17 | 19-60 (35.9) | III |
|  | IBS-C | 13 | 2 | 24-55 (32.3) |  |
|  | IBS-M | 58 | 22 | 18-60 (32.9) |  |
|  | IBS-U | 1 | 0 | 37 |  |
|  | Controls | 25 | 9 | 19-53 (31.6) |  |
| Sweden Sahlgrenska | IBS-D | 47 | 17 | 20-71 (39.5) | II/III |
|  | IBS-C | 26 | 9 | 19-61 (36.1) |  |
|  | IBS-M | 10 | 3 | 21-53 (35.8) |  |
|  | IBS-U | 31 | 9 | 21-64 (35.2) |  |
|  | Controls | 43 | 18 | 20-68 (39.1) |  |
| UK2* | IBS-D | 71 | 25 | 18-72 (45) | III |
|  | IBS-C | 0 | 0 | n.a. |  |
|  | IBS-M | 0 | 0 | n.a. |  |
|  | IBS-U | 0 | 0 | n.a. |  |
|  | Controls | 30 | 84 | 19-67 (36) |  |
| USA1 | IBS-D | 26 | 6 | 20-64 (40.1) |  |
|  | IBS-C | 22 | 3 | 20-58 (39.6) |  |
|  | IBS-M | 9 | 0 | 21-54 (33.2) |  |
|  | IBS-U | 12 | 6 | 20-56 (33) |  |
|  | IBS-A | 16 | 3 | 21-58 (40.8) | II/III |
|  | Controls | 59 | 34 | 19-58 (33.1) |  |
| USA3 | IBS-D | 28 | 8 | 27-91 (54) | III |
|  | IBS-C | 49 | 10 | 26-90 (54) |  |
|  | IBS-M | 46 | 8 | 25-84 (55) |  |
|  | Controls | 48 | 46 | 17-80 (54) |  |

**SD Table 2** Gastrointestinal tissue samples used for expression analysis

^1^Spain (JS), ^2^Germany (MR), ^3^Germany (MGS).

| **Tissue samples** | | | | |
| --- | --- | --- | --- | --- |
| **GI Tissue** | **IBS** | **Age (mean)** | **Controls** | **Age (mean)** |
| Jejunum^1^ | 23 | 22-51 (36) | 26 | 19-53 (31) |
| Ileum^2^ | 16 | 23-82 (48) | 11 | 36-72 (58) |
| Colon^3^ | 21 | 20-74 (48) | 14 | 38-70 (59) |
| Sigma^2^ | 16 | 25-82 (52) | 9 | 36-72 (57) |

**SD Table 3** Summary of genotyped samples from different countries

UK1 (Houghton), Germany1 (Mönnikes), Germany2* (IBS-Net Germany), UK2* (Spiller), UK3 (GLAXO), Ireland* (GC), USA1* (EAM), USA2 (GLAXO), USA3 (GS), Sweden* (MS), Spain* (JS), Greece* (MG), Chile* (CB). *GENIEUR

Additional controls: Germany 2 (PopGen), Sweden (SALT), Spain (INMA).

IBS, Irritable Bowel Syndrome; IBS-C, IBS with constipation; IBS-D, IBS with diarrhea; IBS-M, IBS with mixed bowel habits; IBS-U, undetermined IBS.

| **Genotyped cohorts** | | | | | | | |
| --- | --- | --- | --- | --- | --- | --- | --- |
|  |  | IBS | IBS-C | IBS-D | IBS-M | Controls | Total |
| Discovery | UK1 | 197 | 99 | 98 | 0 | 90 | 287 |
| Validation | Germany1 | 169 | 50 | 119 | 0 | 475 | 644 |
|  | Germany2* | 267 | 25 | 111 | 122 | 1161 | 1428 |
|  | UK2* | 120 | 0 | 120 | 0 | 107 | 227 |
|  | UK3 | 190 | 83 | 107 | 0 | 149 | 339 |
|  | Ireland* | 34 | 3 | 4 | 27 | 50 | 84 |
|  | USA1* | 105 | 26 | 32 | 28 | 93 | 198 |
|  | USA2 | 455 | 185 | 269 | 0 | 487 | 942 |
|  | USA3 | 156 | 59 | 36 | 54 | 93 | 249 |
|  | Sweden* | 196 | 30 | 53 | 87 | 2033 | 2229 |
|  | Spain* | 60 | 0 | 60 | 0 | 1207 | 1267 |
|  | Greece* | 155 | 114 | 35 | 6 | 143 | 298 |
|  | Chile* | 71 | 16 | 14 | 40 | 40 | 111 |
| Pooled | Pooled | 2175 | 690 | 1058 | 364 | 6128 | 8303 |
|  | Mean | 167.31 | 53.08 | 81.38 | 28.00 | 471.38 | 638.69 |

### **SD Table 4** Primers for mutation analysis and genotyping

T_A_, annealing temperature; bp, base pairs

### **SD Table 5** Primers for expression analysis

T_A_, annealing temperature; bp, base pairs

### **SD Table 6** Target sequences for nCounter analysis

*P2 driven isoform; HK, housekeeping gene

| **Gene** | **Accession** | **Position** | **Target sequence** | **Type** |
| --- | --- | --- | --- | --- |
| *ARF1* | NM_001024227.1 | 1371-1470 | CAATTCTGCATGGTC…..TGCTCCCACGGTTCC | HK |
| *SNX17* | NM_014748.2 | 1786-1885 | CTTTCCTTGTCCCCT…..TATTTTGCACAAAGTC | HK |
| *SDHA* | NM_004168.2 | 2223-2322 | AGTACATTGAAGGGA…...CTTGCTTCATTCTTG | HK |
| *HPRT1* | NM_000194.1 | 241-340 | TGTGATGAAGGAGAT…..TTACATCAAAGCACTG | HK |
| *PPIA* | NM_021130.2 | 926-1025 | GGAATATTGAAAATG…...AGGCAGAAGACCACCT | HK |
| *PGK1* | NM_000291.3 | 965-1064 | ATTGTCAAAGACCTA…...GATGAGAATGCCAAGA | HK |
| *UBB* | NM_018955.2 | 796-895 | CACCTGGTCCTGCG…..TAGCCATTTGCCCCAA | HK |
| *SLC6A** | NM_001045.5 | 357-456 | GTCTCCTGGAGGCA…..AAGAAGAACAAATGAGT | Target |

### **SD Table 7** Primers for sequencing and cloning of the promoter P2 region

T_A_, annealing temperature; bp, base pairs

**Patients**

**GlaxoSmithKline cohort**

Two independent cohorts of IBS patients and controls from the UK (termed UK3) and the USA/Canada (termed USA2) from Glaxo, previously termed ‘Belgium samples’^1,2^ were included in this study. Informed consent was obtained from all participants and local ethics committees approved the study protocol. The demographics and clinical characteristics of both cohorts have been reported^3^. The IBS patients were divided into subgroups based on the predominant bowel habit according to the Rome II criteria. All patients were of Caucasian ethnicity^3^.

**Controls**

**PopGen Controls**

Further German control data were taken from the PopGen Controls (<https://www.epidemiologie.uni-kiel.de/node/119>). The rs2020938 SNP was extracted from data of 1,228 individuals. Thirteen SNPs not genotyped in PopGen were imputed into 1,228 PopGen individuals based on 2,577 reference individuals from phase 3 of the 1,000 Genomes Project. IMPUTEv2 was used to impute genotypes (default settings) based on resulting genotype probabilities, and exact genotypes were determined according to the 0.9 threshold rule (calls with uncertainty greater than 0.1 were treated as missing and the rest were treated as hard calls).

**Spain: INMA (INfancia y Medio Ambiente)**

Genotypes of further Spanish controls were taken from the INMA (INfancia y Medio Ambiente – Environment and Childhood) project. This is a network of birth cohorts in Spain that studies the effect of exposure to environmental pollutants in air, water, and diet during pregnancy on early childhood growth and development (<http://www.proyectoinma.org/>)^4^. The study had been approved by the ethical committee of each participating center and written consent was obtained from participating parents. Children from the following sub-cohorts participated in this study: INMA Menorca, INMA Sabadell, and INMA Valencia.

**Sweden: SALT (Screening Across the Lifespan Twin) study**

Additional genotypes of Swedish controls (from the SALT study) were selected from a published IBS GWAS study on a Swedish general population cohort^2^, where 534 IBS patients defined based on Rome criteria and 4,932 asymptomatic controls were included in the analyses. In this study, healthy controls were selected based on reporting no bowel symptoms in an interview. We randomly chose 2,000 Swedish controls for replication studies.

**Cell lines**

Cell lines for expression and functional assays were obtained from the American Tissue Culture Cooperation (ATCC, LGC Standards, Wesel, Germany). These were Caco 2 (human epithelial colorectal adenocarcinoma), Colo 320 (human colorectal adenocarcinoma), HEK293T (human fetal renal cell carcinoma, which are highly transfectable as they carry the large T antigen of Simian virus 40) and SH-SY5Y as well as IMR-32 (both human neuroblastoma)- Cells were cultured as recommend by ATCC.

#### SD Methods

**Sequencing of *SLC6A4* promoter region P2**

***Polymerase chain reaction (PCR)***

PCRs were performed in 12.5 µl volumes containing 25–50 ng of genomic DNA as template, 3.125 pmol of each primer, 5 pmol of each dNTP (MBI Fermentas, St. Leon-Rot, Germany), 1.25 µl HotStarTaq PCR Buffer 10x concentrated (contains Tris-HCl, KCl, (NH_4_)_2_SO_4_, 15 mM MgCl_2_), and 0.625 U (0.125 µl) of HotStarTaq DNA Polymerase (Qiagen, Hilden, Germany).

Thermal cycling was performed in Mastercycler *vapo.protect* thermal cyclers (Eppendorf, Hamburg, Germany). Annealing temperatures (T_A_) and sequences of the promoter region P2 specific *SLC6A4* primers are shown in Supplementary SD Table 4. Cycling conditions were: initial denaturation at 95°C for 15 min followed by 35 cycles of 94°C for 30 s, T_A_ for 30 s, and 72°C for 30 s. The final extension step was at 72°C for 10 min. A 3 µl aliquot of each PCR product was analyzed on a 1.5% agarose gel and imaged using the Quantum-1100 imaging system and the Quantum Capt software (VWR, Darmstadt, Germany).

***Purification and direct sequencing of PCR products***

PCR products were validated by Sanger sequencing as follows: a 5 µl aliquot of the PCR product was treated with 10 U exonuclease I (Fermentas, St- Leon-Rot, Germany) and 1 U thermosensitive alkaline phosphatase (FastAP, Fermentas) in a final volume of 8 µl for 15 min at 37°C followed by inactivation at 80°C for 15 min. 2 µl of the respective *ExoI/FastAP* reaction were used for direct sequencing using the DYE-namic ET Terminator Cycle Sequencing Kit according to the manufacturer’s protocol (GE Healthcare, Little Chalfont, United Kingdom). For sequence analysis, the MegaBACE 1000 sequencer (GE Healthcare) and Geneious software (version 5.3.6 created by Biomatters, available from [http://www.geneious.com)](http://www.geneious.com/) were used.

**Genotyping controls**

**Spain: INfancia y Medio Ambiente**

One thousand and seventy-one children whose parents were white and born in Spain or other European countries and that were not lost during the 4-year follow-up were selected for genotyping. Genome-wide genotyping was performed using the HumanOmni1-Quad v1.0 Beadchip (Illumina) at the Spanish National Genotyping Centre (CEGEN). Genotypes were called using the GeneTrain2.0 algorithm based on HapMap clusters implemented in the GenomeStudio software (Illumina). PLINK was used for genetic data quality control. We applied the following initial quality control thresholds: sample call rate > 98% and/or LRR SD < 0.3 (excluded: N=7: four from Valencia and two from Menorca, 0.7%). Next, we checked sex, relatedness (one duplicate sample and the younger brother of two brother-pairs in the INMA Sabadell cohort were excluded), heterozygosity, and population stratification. Genetic variants were filtered for SNP call rate > 95%, MAF > 1% and Hardy–Weinberg equilibrium (HWE) *P*-value > 1.10E-6. The final genetic data set consisted of 1,061 subjects from INMA Sabadell (N = 396), INMA Valencia (N = 349) and INMA Menorca (N = 316) and 817.131 QCed SNPs in b36 and + strand.

Imputation was performed with IMPUTEv2 using a cosmopolitan reference panel from the 1000 Genomes project (rel March 2012). After imputation, the database consisted of 39.346-413 SNPs (38.546.049 imputed and 800.364 genotyped).

**Sweden SALT**

The SALT individuals were genotyped using the Illumina OmniExpress platform and basic quality control metrics were applied at both SNP and sample levels. Briefly, SNPs with call rates < 95% and/or HWE *p* < 10^−5^ were excluded. Samples were removed if: 1) call rates were < 98%, 2) genotype-imputed sex did not match the phenotype-reported sex, 3) they possessed an extreme heterozygosity rate (out of 3*SD of mean heterozygosity rate), and 4) they were related to other samples (PI-HAT > 0.1875), in which case the one with the highest missing rate in a related pair was removed.

Genome-wide imputation was then performed in two steps using Shapeit2 for phasing and IMPUTEv2 for imputation^5^. The 1000 genomes cosmopolitan population phase3 was used as a reference panel. Before imputation, all genotypes were aligned on the same strand with the reference panel using the “Genotype harmonizer” tool^6^. All SNPs that failed to align on the strand were excluded from further analyses. After imputation, we only kept SNPs with high imputation quality (info value of 0.8 and higher).

**RNA extraction and reverse transcription**

To quantify gene expression and to correlate differential expression driven by the SNP rs2020938 in P2, total RNA was extracted from various cell lines (HEK293T, Caco 2, Colo 320, SH-SY5Y, IMR-32) and from small and large intestine tissues (jejunum, ileum, colon, sigma) of IBS patients and control individuals using TRIZOL. One μg of total RNA was reverse transcribed into complementary DNA (cDNA) using the Superscript III-First-Strand-Synthesis-System (Invitrogen) as recommended by the manufacturer.

**RT-PCR**

Conventional RT-PCR from reverse transcribed cDNA was performed in 12.5 µl volumes containing 1–2 µl cDNA as template, 3.125 pmol of each primer, 5 pmol of each dNTP (MBI Fermentas), 1.25 µl HotStarTaq PCR Buffer 10x concentrated (contains Tris-HCl, KCl, (NH_4_)_2_SO_4_, 15 mM MgCl_2_), and 0.625 U (0.125 µl) of HotStarTaq DNA Polymerase (Qiagen).

Thermal cycling was performed in Mastercycler *vapo.protect* thermal cyclers (Eppendorf, Hamburg, Germany). Annealing temperatures (T_A_) and sequences of specific *SLC6A4* expression primers are shown in Supplementary SD Table 5. Cycling conditions were: 15 min 95°C, followed by 40 cycles of 30 sec 94°C, 30 sec T_A_, 30 sec 72°C, and a final elongation for 10 min at 72°C. A 3 µl aliquot of each PCR product was analyzed on a 1.5 % agarose gel and imaged using the Quantum-1100 Imaging System and the Quantum Capt software (VWR).

**Quantitative PCR (qPCR)**

Relative gene expression was analyzed by qPCR on a 7500 Fast Real-Time PCR System (Applied Biosystems, Foster City, California) according to the manufacturer’s instructions. Forward and reverse primers and TaqMan probes specific for *SLC6A4* P1 and P2 promoters were designed according to standard guidelines and their relative efficiency tested as described in qPCR Application Guide Experimental Overview, Protocol, Troubleshooting. Third Edition (<https://www.gene-quantification.de/idt-miqe-qpcrguide-2010.pdf>). Primer sequences are specified in Supplementary SD Table 5. All values were normalized to 18 S RNA (Applied Biosystems, TaqMan Assay ID: Hs99999901_s1). Each sample, including sterile distilled water as a negative control, was run in triplicate and data were analyzed by the 2-ΔΔCt method with correction for primer efficiency.

**nCounter analysis**

The jejunum was the only tissue with robust P2-driven SERT expression, so nCounter targeted gene expression analysis (nanoString Technologies, Seattle, WA, USA) was carried out on jejunal samples. The nCounter technology allows for multiplexed gene expression analysis based on simultaneous hybridization and digital quantification of fluorescently labeled oligonucleotide probes^7^. A reporter probe which carries a fluorescent barcode, a biotinylated capture probe (elements tag set) that immobilizes the complex for data collection, and two target gene-specific oligonucleotide probes A+B (probe set) are necessary to detect each transcript (Supplementary SD Table 6).

All RNA samples were quantified by using the Qubit^TM^ RNA HS assay kit and quality was controlled using the Agilent RNA6000 Nanokit on an Agilent 2100 Bioanalyzer system. Qualifying samples were subjected to nCounter analysis as recommended by the manufacturer. In brief, one hundred ng of total RNA were used as input material for Elements probe set hybridization at 65°C. The experiment was read out using the SPRINT^TM^ Profiler from nanoString Technologies. Data were normalized and evaluated using the nSolver Analysis Software (version 2.0) provided by nanoString Technologies (<https://www.nanostring.com/products/analysis-software/nsolver>). Stably expressed reference genes were chosen for normalization based on the Normfinder method^8^.

**Transfections**

To investigate the functional effect of the most contrasting major and minor P2 promoter haplotypes on gene transcription, the human embryonic kidney cell line HEK292T, the human neuroblastoma cell line SH-SY5Y, the colorectal carcinoma cell lines Caco 2 and Colo 320 cells were seeded into 6-well plates with approximately 40–70% cell density in Opti-MEM I Reduced Serum Media + 10 % FBS (both Thermo Fisher Scientific) one day prior to transfection. All cell lines but SH-SY5Y were transfected with Polyethylenimin (PEI, Sigma Aldrich) applying a ratio of 1:4 (1 μg DNA: 4 μg PEI). SH-SY5Y cells were transfected using Lipofectamine 2000 (Thermo Fisher Scientific) as recommended by the manufacturer. 24 hours post-transfection, cells were lysed in 100 μl 1x Passive Lysis buffer (Promega, Madison, Wisconsin) per reaction. Lysed cells were frozen at -80°C for one hour and dissolved in lysis buffer prior to luciferase assay.

**Luciferase reporter assays**

For luciferase assays, *SLC6A* P2 promoter constructs were made using pGL3 basic vectors (Promega). The two major haplotype version based promoters were cloned upstream via *XhoI* and *HindIIII* (Supplementary SD Table 7). Reporter gene constructs used were pGL3 basic vector (with and without upstream P2 region insert), the pGL3 control vector (both Promega), and PRL-TK-Vector (reference construct for normalization). 25 μl of each cell lysate was placed in each well of a 96-well plate as input material for the luciferase assay. The assay was performed using the Dual-Luciferase Reporter Assay Systems (Promega) on a Lucy2 luminometer (Rosys Anthos Mikrosysteme, Krefeld, Germany) as recommended by the manufacturer.

**Transcription factor binding site prediction**

Transcription factor binding sites of the promoter P2 region containing the SNPs including rs2020938 linked in the major and minor haplotype were compared using the online tool ePOSSUM2 (<https://www.mutationdistiller.org/ePOSSUM2/>). The input sequences were as follows.

Risk – major haplotype: aaagcctatcaagcctagttgagggcccaaggaagggggaagtggggggaggccaaggccaggcagtagcataaatggtgagcagggtgaggttatggagatcgctcttgtcagaattcatatccttctgagtggtgtttgcattcttgagcctggggtgggtggtggagggcccaacaagtgtggtacagcccaagacg ;

Protective - minor haplotype : aaagcctatcaagcctagttgagggccgaaggaagggggaagtggggggaggccaaggccaggcagtagcataaatggtgagcagggtgaggttatggagaccgctcttgtcagaattcatatccttctgagtggtgtttgcattcttgagcctggggtgggtggcggagggcccaacaagtgcggtacagcccaagacg

This tool predicts the impact of DNA variants on transcription factor binding. ePOSSUM2 supports more than 1,000 different binding models for 247 human transcription factors. The models were pooled from various publicly accessible data sources, such as [CIS-BP](http://cisbp.ccbr.utoronto.ca/), [Hocomoco](http://hocomoco11.autosome.ru/), [hPDI](http://bioinfo.wilmer.jhu.edu/PDI/), [JASPAR](http://jaspar.genereg.net/), and [Jolma (2013)](https://linkinghub.elsevier.com/retrieve/pii/S0092-8674(12)01496-1). Overall, 103 [transcription factor flexible models](https://journals.plos.org/ploscompbiol/article?id=10.1371/journal.pcbi.1003214) are included. For each binding model, ePOSSUM2 constructs a Bayes classifier and reports the likelihood that a variant leads to the gain or loss of a transcription factor binding site. Because several binding models detected experimentally verified sites poorly, a negative or positive predictive value is reported. ePOSSUM2 was developed in 2018 at the [Berlin Institute of Health (BIH)](https://www.bihealth.org/en/research/core-facilities/bioinformatics/) and [Charité – Universitätsmedizin Berlin](https://www.charite.de/en/) by Robin Steinhaus, [Peter N. Robinson](https://www.jax.org/research-and-faculty/faculty/peter-robinson), and [Dominik Seelow](http://mitonet.charite.de/team/dominikseelow.html). ePOSSUM2 is an update of the [ePOSSUM software](http://mutationtaster.charite.de/ePOSSUM/).

In order to collect further evidence and validate ePOSSUM predictions, we explored of ChIP Atlas data (<https://chip-atlas.org/search>) for transcription factors binding in the corresponding promoter region of *SLC6A4*.

**Ingenuity pathway analysis (IPA)**

Pathway and network analysis was performed using IPA software (Ingenuity® Systems, https://www.qiagenbioinformatics.com/). IPA integrates gene data sets with all the biological information available in public databases to predict the functional biological context (biological functions and signaling pathways). For network analysis, IPA provides a score according to the fit of supplied genes and the list of biological functions involved. Core analysis was performed on the 52 transcription factors predicted by ePOSSUM2.

**Statistical analysis for luciferase assays and qPCR analysis**

Two-tailed parametric tests were used as appropriate (unpaired *t*-test, one-way ANOVA followed by Bonferroni correction post-hoc test) using GraphPad Prism 5.0 software (GraphPad Software, Inc., La Jolla; California). A Mann-Whitney *U* test and an unpaired t-test with Welch's correction were applied when data did not follow a normal distribution, as indicated in the figure legends.

Relationships between clinical features (bowel movement, stool form) and qPCR-based mRNA expression levels were assessed by Spearman’s correlation rho. Data are expressed as mean ± standard error of the mean, unless stated otherwise. *P*-values of < 0.05 (**P*< 0.05) were considered statistically significant.

Luciferase data analysis was performed using GraphPad Prism 8.4.2 software applying unpaired multiple t tests of all data. Discovery was determined using the two-stage linear step-up procedure of Benjamini, Krieger, and Yekutieli.

#### SD Results

#### *SLC6A4* expression analysis in different cell lines

To select appropriate cell lines for functional follow-up studies of the variant promoter P2 haplotypes, we assessed the expression profile of S*LC6A4* in various cell lines (SH-SY5Y, IMR-32, Caco 2, HEK293T, and Colo 320 cells; Supplementary SD Figure 2). For this purpose, we isolated total RNA and generated cDNAs for RT-PCR analysis. All analyzed cell lines expressed *SLC6A4* and were therefore all selected for functional analysis of the major and minor haplotypes of promoter P2 by luciferase reporter assays except for IMR-32.

**SD Results Tables**

**SD Table 8** Detected variants in promoter region P2, *GnomAD, na not annotated

### **SD Table 9** Test for deviation from Hardy–Weinberg Equilibrium (*P*-value) in patient and control samples

| ***SLC6A4*** | **rs2020938** | | | | |
| --- | --- | --- | --- | --- | --- |
| Study cohort | IBS overall | IBS-C | controls | female IBS-C | female controls |
| UK1 | 0.31 | 0.027 | 0.26 | 0.041 | 0.74 |
| Germany1 | 0.39 | 1.00 | 1.00 | 1.00 | 1.00 |
| Germany2 | 0.33 | 0.50 | 0.45 | 0.54 | 0.57 |
| UK2+3 | 0.63 | 0.73 | 1.00 | 0.70 | 0.76 |
| Ireland | 0.56 | 1.00 | 0.42 | 1.00 | 0.65 |
| USA1 | 1.00 | 1.00 | 0.43 | 1.00 | 1.00 |
| USA2 | 0.88 | 0.82 | 1.00 | 1.00 | 0.75 |
| USA3 | 0.47 | 0.064 | 0.49 | 0.17 | 1.00 |
| Sweden | 0.43 | 1.00 | 1.00 | 1.00 | 0.30 |
| Spain | 1.00 | n.a. | 0.22 | n.a. | 0.39 |
| Greece | 1,65E-12 | 1,65E-12 | 0.065 | 1,68E-12 | 0.17 |
| Chile | 0.74 | 1.00 | 1.00 | 1.00 | 1.00 |

### **SD Table 10** Ct values of qPCR data *SLC6A4* P1/P2 driven isoforms

| **Sample code** | **Group** | **Tissue** | **18s Ct** | ***SLC6A4* P1 Ct** | ***SLC6A4* Ct** |
| --- | --- | --- | --- | --- | --- |
| ER-MK-MR-003 | IBS | Ileum | 16,57833337 | 35,50196707 | 24,89783242 |
| ER-MK-MR-004 | IBS | Ileum | 18,76806721 | 38,74713988 | 27,17786836 |
| ER-MK-MR-007 | IBS | Ileum | 15,78976814 | 33,12196345 | 23,75244634 |
| ER-MK-MR-009 | IBS | Ileum | 15,1460357 | 32,19460727 | 22,81192444 |
| ER-MK-MR-010 | IBS | Ileum | 15,46972902 | 33,072317 | 23,55361965 |
| ER-MK-MR-011 | IBS | Ileum | 15,3973569 | 35,74640249 | 25,45528824 |
| ER-MK-MR-012 | IBS | Ileum | 15,69719769 | 34,74001429 | 24,84114996 |
| ER-MK-MR-013 | IBS | Ileum | 16,87498135 | 35,16516928 | 25,75574305 |
| ER-MK-MR-015 | IBS | Ileum | 15,98474015 | 34,61576191 | 23,25792871 |
| ER-MK-MR-017 | IBS | Ileum | 15,62578989 | 34,0614149 | 24,18704425 |
| ER-MK-MR-019 | IBS | Ileum | 15,75802725 | 35,07133089 | 23,30192139 |
| ER-MK-MR-020 | IBS | Ileum | 16,62464062 | 34,38156678 | 23,74253981 |
| ER-MK-MR-021 | IBS | Ileum | 16,23376757 | 32,30141317 | 23,7278778 |
| ER-MK-MR-025 | IBS | Ileum | 17,22877481 | 35,8002207 | 25,25428307 |
| ER-MK-MR-026 | IBS | Ileum | 16,24136959 | 35,13770016 | 23,90798373 |
| ER-MK-MR-027 | IBS | Ileum | 15,73517447 | 33,38682527 | 23,23383261 |
| ER-MK-MR-028 | IBS | Ileum | 16,32410269 | 34,58312108 | 23,7072518 |
| ER-MK-MR-001 | Healthy control | Ileum | 15,36049295 | 35,90217299 | 27,2274689 |
| ER-MK-MR-006 | Healthy control | Ileum | 15,61590827 | 33,93056589 | 23,76490999 |
| ER-MK-MR-008 | Healthy control | Ileum | 15,29043582 | 33,64500865 | 24,22376062 |
| ER-MK-MR-016 | Healthy control | Ileum | 15,44596624 | 32,98832515 | 23,42277352 |
| ER-MK-MR-022 | Healthy control | Ileum | 16,31101889 | 33,13384236 | 23,38881028 |
| ER-MK-MR-023 | Healthy control | Ileum | 16,25127578 | 34,56713914 | 23,66936656 |
| ER-MK-MR-024 | Healthy control | Ileum | 16,45577012 | 38,22063153 | 25,88062652 |
| ER-MK-MR-029 | Healthy control | Ileum | 16,01129456 | 33,42104593 | 23,33013675 |
| ER-MK-MR-030 | Healthy control | Ileum | 15,93677023 | 34,05102097 | 23,43950417 |
| BCN2 | IBS | Jejunum | 18,34616089 | 39,73058166 | 30,81976709 |
| BCN3 | IBS | Jejunum | 17,23204803 | 37,34487938 | 28,94345808 |
| BCN5 | IBS | Jejunum | 17,95299339 | 38,33751658 | 30,32406309 |
| BCN6 | IBS | Jejunum | 17,66653442 | 37,72347639 | 30,7431139 |
| BCN7 | IBS | Jejunum | 16,87281036 | 37,535122 | 28,7124546 |
| BCN16 | IBS | Jejunum | 17,40193558 | 35,97644571 | 28,0237678 |
| BCN17 | IBS | Jejunum | 17,0983696 | 35,45454652 | 27,2499728 |
| BCN18 | IBS | Jejunum | 17,74133682 | 34,39541101 | 27,35790818 |
| BCN19 | IBS | Jejunum | 17,19239807 | 36,32251025 | 28,4785045 |
| BCN42 | IBS | Jejunum | 17,55171204 | 36,59863221 | 28,46095552 |
| BCN43 | IBS | Jejunum | 17,61321068 | 35,55451009 | 27,49397446 |
| BCN44 | IBS | Jejunum | 17,00183296 | 34,56787078 | 27,78468358 |
| BCN45 | IBS | Jejunum | 18,14543343 | 37,32597404 | 29,11618248 |
| BCN46 | IBS | Jejunum | 19,03618622 | undetermined | 29,4530488 |
| BCN47 | IBS | Jejunum | 18,01171875 | 35,10915844 | 27,05952716 |
| BCN48 | IBS | Jejunum | 16,81947327 | 35,40755294 | 27,68018541 |
| BCN49 | IBS | Jejunum | 17,3154335 | 34,56916434 | 27,29516354 |
| BCN50 | IBS | Jejunum | 17,83892822 | 37,95594248 | 28,89515524 |
| BCN51 | IBS | Jejunum | 18,1 | 35,34533878 | 29,22341294 |
| BCN52 | IBS | Jejunum | 18,32463455 | 38,31718656 | 29,75942126 |
| BCN53 | IBS | Jejunum | 18,49115372 | 37,71374177 | 29,60427638 |
| BCN54 | IBS | Jejunum | 17,05583572 | 34,30906855 | 27,01303968 |
| BCN55 | IBS | Jejunum | 17,57375336 | 35,00560603 | 28,07346943 |
| BCN56 | IBS | Jejunum | 17,06829071 | 37,18562865 | 30,42199954 |
| BCN57 | IBS | Jejunum | 17,04096794 | 33,59088479 | 27,14901846 |
| BCN58 | IBS | Jejunum | 16,96086884 | 35,70972135 | 27,49031071 |
| BCN59 | IBS | Jejunum | 17,35703278 | 36,67221818 | 27,5330794 |
| BCN60 | IBS | Jejunum | 16,87817001 | 38,15106697 | 27,78926574 |
| BCN61 | IBS | Jejunum | 16,86527443 | 35,32750925 | 27,15187509 |
| BCN62 | IBS | Jejunum | 16,55615997 | 35,38464389 | 27,88648716 |
| BCN63 | IBS | Jejunum | 17,13666344 | 35,46373373 | 28,07083258 |
| BCN64 | IBS | Jejunum | 16,91453362 | 35,33075682 | 27,13665483 |
| BCN70 | IBS | Jejunum | 18,97595406 | 37,41222347 | 30,45523216 |
| BCN71 | IBS | Jejunum | 18,5400238 | 39,77472022 | 30,72117842 |
| BCN72 | IBS | Jejunum | 16,72651863 | 36,78650715 | 27,81226619 |
| BCN73 | IBS | Jejunum | 19,53974533 | undetermined | 31,12689689 |
| BCN74 | IBS | Jejunum | 17,49632454 | 38,26791168 | 28,55567605 |
| BCN28 | Healthy control | Jejunum | 16,84288406 | 36,49087586 | 28,4539809 |
| BCN34 | Healthy control | Jejunum | 17,50685883 | 37,7218735 | 29,86843379 |
| BCN35 | Healthy control | Jejunum | 17,85357285 | 36,46522095 | 28,58128621 |
| BCN36 | Healthy control | Jejunum | 17,57317352 | 35,45570037 | 28,26737634 |
| BCN37 | Healthy control | Jejunum | 17,87538147 | 37,72672186 | 27,61536111 |
| BCN38 | Healthy control | Jejunum | 17,77909851 | 36,77850621 | 28,74544825 |
| BCN39 | Healthy control | Jejunum | 16,60348129 | 35,62351994 | 27,77497335 |
| BCN40 | Healthy control | Jejunum | 16,90099335 | 34,6715074 | 28,05497559 |
| BCN41 | Healthy control | Jejunum | 18,10161018 | 35,58449196 | 28,41336675 |
| BCN68 | Healthy control | Jejunum | 16,5283432 | 33,76212561 | 26,99324395 |
| BCN69 | Healthy control | Jejunum | 17,0928421 | 35,06656377 | 26,82039283 |
| ER-MK-MR-007 | IBS | Sigma | 17,44391868 | 34,29 | 35,26 |
| ER-MK-MR-009 | IBS | Sigma | 15,43060381 | Undertemined | 32,51101748 |
| ER-MK-MR-010 | IBS | Sigma | 15,51610788 | 36,32457542 | 33,01600647 |
| ER-MK-MR-011 | IBS | Sigma | 16,34921776 | Undertemined | 35,95563889 |
| ER-MK-MR-012 | IBS | Sigma | 16,68834373 | Undertemined | 37 |
| ER-MK-MR-013 | IBS | Sigma | 16,40824573 | 36,99 | 35,12139384 |
| ER-MK-MR-014 | IBS | Sigma | 16,13374419 | Undertemined | 36,94646962 |
| ER-MK-MR-015 | IBS | Sigma | 16,00693323 | 35,41 | 34,66649628 |
| ER-MK-MR-017 | IBS | Sigma | 16,02373634 | 36,55 | 37,24346542 |
| ER-MK-MR-018 | IBS | Sigma | 16,59000327 | Undertemined | 34,03622818 |
| ER-MK-MR-019 | IBS | Sigma | 15,55161112 | 37,1 | 34,81573232 |
| ER-MK-MR-021 | IBS | Sigma | 16,00698701 | Undertemined | 35,36591085 |
| ER-MK-MR-025 | IBS | Sigma | 16,27040304 | 36,2 | 31,30365817 |
| ER-MK-MR-008 | Healthy control | Sigma | 15,87784914 | 35,15 | 31,0500164 |
| ER-MK-MR-016 | Healthy control | Sigma | 16,38361487 | Undertemined | 31,99977748 |
| ER-MK-MR-022 | Healthy control | Sigma | 17,29106473 | 36,48316193 | 34,16260529 |
| ER-MK-MR-023 | Healthy control | Sigma | 16,13667951 | 35,6 | 33,66689555 |
| ER-MK-MR-024 | Healthy control | Sigma | 17,29880359 | Undertemined | 32,77197393 |
| 2 | IBS | Colon | 18,29960962 | Undetermined | 32,87808355 |
| 5 | IBS | Colon | 17,20583263 | Undetermined | 35,04758708 |
| 6 | IBS | Colon | 16,3521575 | Undetermined | 31,46317927 |
| 13 | IBS | Colon | 17,55937758 | 36,94776535 | 32,295976 |
| 14 | IBS | Colon | 16,88134367 | Undetermined | 30,15899531 |
| 19 | IBS | Colon | 17,08260609 | 36,56434441 | 31,34770012 |
| 20 | IBS | Colon | 16,78699822 | Undetermined | 31,73283323 |
| 22 | IBS | Colon | 19,54240328 | Undetermined | 32,46275965 |
| 23 | IBS | Colon | 17,29031609 | 36,63349724 | 30,70471509 |
| 24 | IBS | Colon | 17,01536734 | Undetermined | 36,97 |
| 26 | IBS | Colon | 17,93946522 | 35,74 | 30,5747172 |
| 27 | IBS | Colon | 16,8949637 | Undetermined | 30,973032 |
| 28 | IBS | Colon | 17,35046852 | Undetermined | 30,70275688 |
| 29 | IBS | Colon | 16,4955049 | 35,54 | 30,66182264 |
| 30 | IBS | Colon | 17,31728934 | 35,84172058 | 30,76026217 |
| 33 | IBS | Colon | 17,05520735 | Undetermined | 32,4541022 |
| 34 | IBS | Colon | 17,53008811 | Undetermined | 31,86995443 |
| 35 | IBS | Colon | 18,39402905 | 36,37 | 30,52995173 |
| 36 | IBS | Colon | 16,67222367 | 36,94 | 30,51552773 |
| 38 | IBS | Colon | 17,07300162 | Undertemined | 30,8369058 |
| 39 | IBS | Colon | 18,57020071 | Undertemined | 32,13900566 |
| 3 | Healthy control | Colon | 16,81724649 | Undetermined | 31,96453094 |
| 7 | Healthy control | Colon | 17,34802874 | Undetermined | 31,65384229 |
| 8 | Healthy control | Colon | 16,91298886 | 35,25 | 33,52826436 |
| 9 | Healthy control | Colon | 17,08674982 | 36,3321209 | 31,08000628 |
| 10 | Healthy control | Colon | 16,8024042 | Undetermined | 31,54023679 |
| 15 | Healthy control | Colon | 17,20890205 | Undetermined | 32,75512441 |
| 16 | Healthy control | Colon | 17,75628429 | Undetermined | 36,59430122 |
| 17 | Healthy control | Colon | 17,18627134 | Undetermined | 33,67042414 |
| 18 | Healthy control | Colon | 17,38047673 | Undetermined | 31,8262469 |
| 21 | Healthy control | Colon | 17,08621864 | Undetermined | 30,93513743 |
| 25 | Healthy control | Colon | 16,57395005 | 35,83358256 | 30,62000529 |
| 31 | Healthy control | Colon | 17,56393995 | Undetermined | 30,31180064 |
| 32 | Healthy control | Colon | 17,0698365 | 36,83 | 32,20366542 |
| 37 | Healthy control | Colon | 18,23812589 | 35,62 | 30,45488739 |

**SD Table 11** ePOSSUM2 predicted transcription factors with gained binding sites in the polymorphic minor haplotype of the P2 promoter region and corresponding ChIP atlas data. TFs not included in ChIP Atlas data are indicated in grey.

**SD Table 12** GTEx eQTL data rs2020938 (Single-Tissue eQTLs for chr17_30222791_C_T_b38; Data Source: GTEx Analysis Release V8 (dbGaP Accession phs000424.v8. p2))

**SD Figures**

**
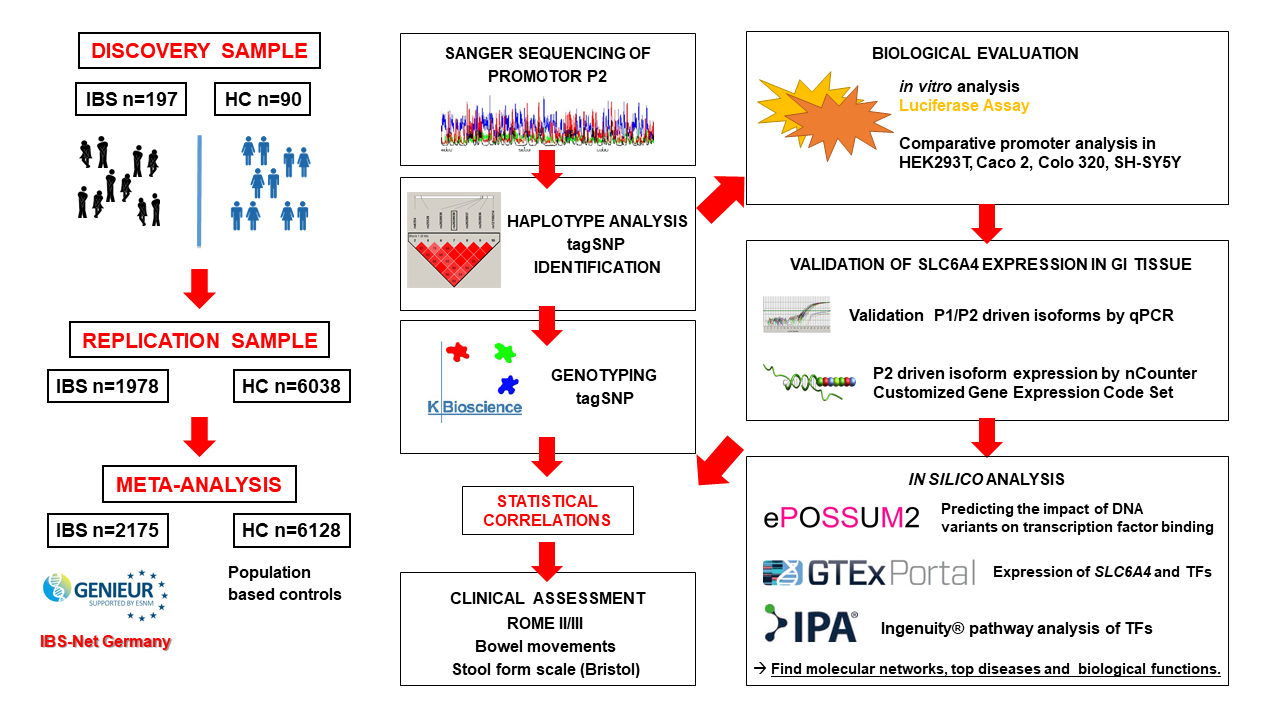
**

**SD Figure 1 Summary of experimental design.** Workflow of the analysis performed on biospecimens of human subjects and complementing *in vitro* and *in silico* analyses into the functional relevance of detected variants.

**
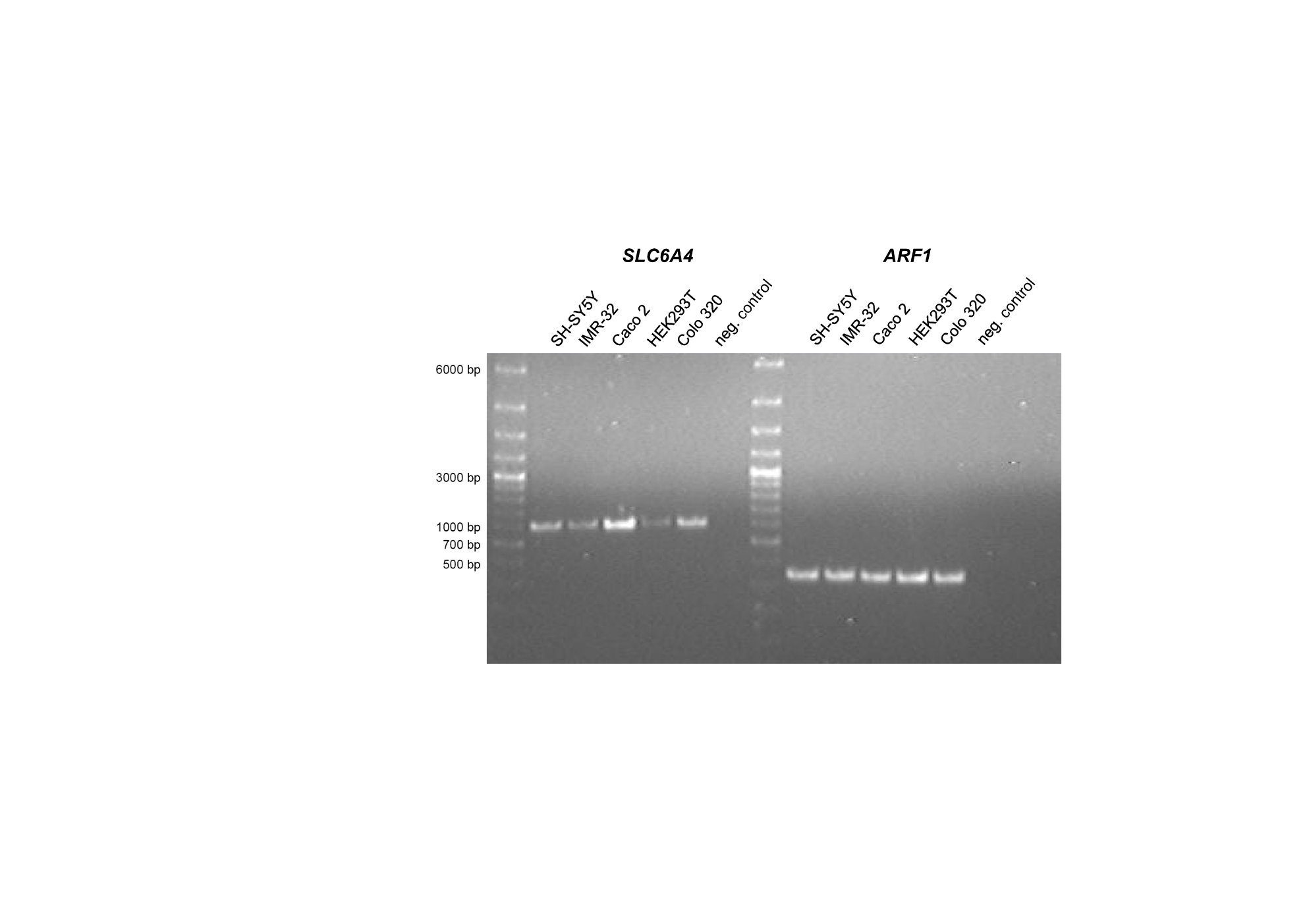
**

**SD Figure 2** *SLC6A4* expression validation in SH-SY5Y, IMR-32, Caco 2, HEK293T, and Colo 320 cells. *ARF1* was included as positive control for cDNA integrity. Marker: Gene Ruler™1kb DNA ladder (MBI Fermentas). Note: IMR-32 was not further included in analyses.

#### SD References

1. Wohlfarth C, Schmitteckert S, Hartle JD, et al. miR-16 and miR-103 impact 5-HT4 receptor signalling and correlate with symptom profile in irritable bowel syndrome. *Sci Rep*. Oct 31 2017;7(1):14680. <https://doi.org10.1038/s41598-017-13982-0>.

2. Ek WE, Reznichenko A, Ripke S, et al. Exploring the genetics of irritable bowel syndrome: a GWA study in the general population and replication in multinational case-control cohorts. *Gut*. Nov 2015;64(11):1774-82. <https://doi.org10.1136/gutjnl-2014-307997>.

3. Wouters MM, Lambrechts D, Knapp M, et al. Genetic variants in CDC42 and NXPH1 as susceptibility factors for constipation and diarrhoea predominant irritable bowel syndrome. *Gut*. Jul 2014;63(7):1103-11. <https://doi.org10.1136/gutjnl-2013-304570>.

4. Guxens M, Ballester F, Espada M, et al. Cohort Profile: the INMA--INfancia y Medio Ambiente--(Environment and Childhood) Project. *Int J Epidemiol*. Aug 2012;41(4):930-40. <https://doi.org10.1093/ije/dyr054>.

5. van Leeuwen EM, Kanterakis A, Deelen P, et al. Population-specific genotype imputations using minimac or IMPUTE2. *Nat Protoc*. Sep 2015;10(9):1285-96. <https://doi.org10.1038/nprot.2015.077>.

6. Deelen P, Bonder MJ, van der Velde KJ, et al. Genotype harmonizer: automatic strand alignment and format conversion for genotype data integration. *BMC Res Notes*. Dec 11 2014;7:901. <https://doi.org10.1186/1756-0500-7-901>.

7. Geiss GK, Bumgarner RE, Birditt B, et al. Direct multiplexed measurement of gene expression with color-coded probe pairs. *Nat Biotechnol*. Mar 2008;26(3):317-25. <https://doi.org10.1038/nbt1385>.

8. Vandesompele J, De Preter K, Pattyn F, et al. Accurate normalization of real-time quantitative RT-PCR data by geometric averaging of multiple internal control genes. *Genome Biol*. Jun 18 2002;3(7):RESEARCH0034.

**Acknowledgements**

We thank Dr. Theadore Ptak, Renee Henry, Ellen Goldstein, Cindy Lee, Deborah Roach, Jacqueline Rabuzin and Elizabeth Crosland (Toronto Digestive Disease Associates (TDDA Inc.), Toronto, Ontario, Canada), Dr. Mark Silverberg and Lori Baladjay (Mount Sinai Hospital, Toronto, Ontario, Canada), Dr. Yehuda Ringel, Dr. Robert Sandler, Alesia N. Aileo, Sarah Causey and Sarah Yeskel (University of North Carolina at Chapel Hill, NC, USA) as well as the staff at Ersta Hospital (Stockholm, Sweden) for collecting samples and acquiring data and Dr. Rachel Gibson and Dr. George Dukes (GlaxoSmithKline) for logistic support as well as for patient recruitment and clinical data collection.

Thanks to Milagros Gallart, Adoración Nieto, Laura Hernández and Sara Méndez from Hospital Universitari Vall d’Hebron for their invaluable assistance in the performance of jejunal biopsies. GB is supported by a grant from the Flanders Research Foundation (FWO, Odysseus). MW was supported by a FWO postdoctoral research fellowship. MD was funded by the Swedish Research Council (VR). APC Microbiome Ireland is funded by Science Foundation Ireland (SFI), through the Irish Government’s National Development Plan. The authors and their work were supported by SFI (grant number SFI/12/RC/2273 P2) and by the Health Research Board through Health Research Awards (grant no HRA_POR/2011/23; TGD, JFC, and GC). MS is supported by the Swedish Medical Research Council (grants 13409, 21691 and 21692), the Marianne and Marcus Wallenberg Foundation, and the University of Gothenburg, Centre for Person-Centred Care (GPCC), Sahlgrenska Academy, University of Gothenburg and by the Faculty of Medicine, University of Gothenburg; MV and JS are supported by Fondo de Investigación Sanitaria and CIBERehd, Instituto de Salud Carlos III, Subdirección General de Investigación Sanitaria, Ministerio de Economía y Competitividad (PI13/00935, PI14/00994); CM is supported by Instituto de Salud Carlos III, Subdirección General de Investigación Sanitaria, Ministerio de Ciencia, Innovación y Universidades (CP18/00116). The UK Nottingham cohort was supported by a Research for Patient Benefit grant and salary support for Dr. Garsed from the Nottingham Digestive Diseases Biomedical Research Unit. The Chilean Santiago cohort was supported by FONDECYT No11121527, No1181699.

The Genotype-Tissue Expression (GTEx) Project was supported by the [Common Fund](https://commonfund.nih.gov/GTEx) of the Office of the Director of the National Institutes of Health as well as by NCI, NHGRI, NHLBI, NIDA, NIMH, and NINDS. The data used for the analyses described in this manuscript were obtained from the GTEx portal (Release v08 on 26 January 2021).
